# Spike-Driven Glutamate Electrodiffusion Triggers Synaptic Potentiation via a Homer-Dependent mGluR-NMDAR Link

**DOI:** 10.1016/j.neuron.2012.11.026

**Published:** 2013-02-06

**Authors:** Sergiy Sylantyev, Leonid P. Savtchenko, Yaroslav Ermolyuk, Piotr Michaluk, Dmitri A. Rusakov

**Affiliations:** 1 UCL Institute of Neurology, University College London, Queen Square, London WC1N 3BG, UK

## Abstract

Electric fields of synaptic currents can influence diffusion of charged neurotransmitters, such as glutamate, in the synaptic cleft. However, this phenomenon has hitherto been detected only through sustained depolarization of large principal neurons, and its adaptive significance remains unknown. Here, we find that in cerebellar synapses formed on electrically compact granule cells, a single postsynaptic action potential can retard escape of glutamate released into the cleft. This retardation boosts activation of perisynaptic group I metabotropic glutamate receptors (mGluRs), which in turn rapidly facilitates local NMDA receptor currents. The underlying mechanism relies on a Homer-containing protein scaffold, but not GPCR- or Ca^2+^-dependent signaling. Through the mGluR-NMDAR interaction, the coincidence between a postsynaptic spike and glutamate release triggers a lasting enhancement of synaptic transmission that alters the basic integrate-and-spike rule in the circuitry. Our results thus reveal an electrodiffusion-driven synaptic memory mechanism that requires high-precision coincidence detection suitable for high-fidelity circuitries.

## Highlights

► A single action potential decelerates diffusion escape of released glutamate ► Slowing down glutamate diffusion enhances activation of perisynaptic group I mGluRs ► Activation of mGluRs boosts local NMDAR currents in a Homer1-dependent manner ► Postsynaptic spike-release pairing induces NMDAR- and mGluR-dependent LTP

## Teaser

Sylantyev et al. find that electric fields of postsynaptic spikes retard escape of glutamate from the synaptic cleft thus boosting activation of perisynaptic metabotropic receptors in cerebellar synapses. This leads to enhancement of synaptic NMDA receptor current leading to long-term potentiation of transmission.

## Introduction

Electric currents flowing through synaptic receptor channels can give rise to substantial electric fields inside the narrow synaptic cleft (Savtchenko and Rusakov, 2007), a phenomenon predicted analytically decades ago by Eccles and Jaeger (1958). Because some common neurotransmitters, such as glutamate or acetylcholine, bear an electric charge at physiological pH, such fields should affect their escape from the cleft, thus impinging on the waveform of synaptic currents (Clements, 1996; Nielsen et al., 2004; Rusakov and Kullmann, 1998), hence signal integration in the brain (London and Häusser, 2005). We previously found that synaptic currents could indeed influence intracleft glutamate diffusion at CA3-CA1 synapses in the hippocampus (Sylantyev et al., 2008). However, this phenomenon could only reveal itself as a slowdown of the EPSC decay, or an increase in the intracleft concentration of released glutamate, upon sustained postsynaptic depolarization above zero. Such depolarization is unlikely to happen in vivo. In addition, the accurate interpretation of remote synaptic events using somatic recordings in large CA1 pyramidal cells could be complicated by space-clamp errors (Williams and Mitchell, 2008). The adaptive physiological significance of electric fields interacting with glutamate inside the synaptic cleft remains therefore uncertain.

**Figure 1:**
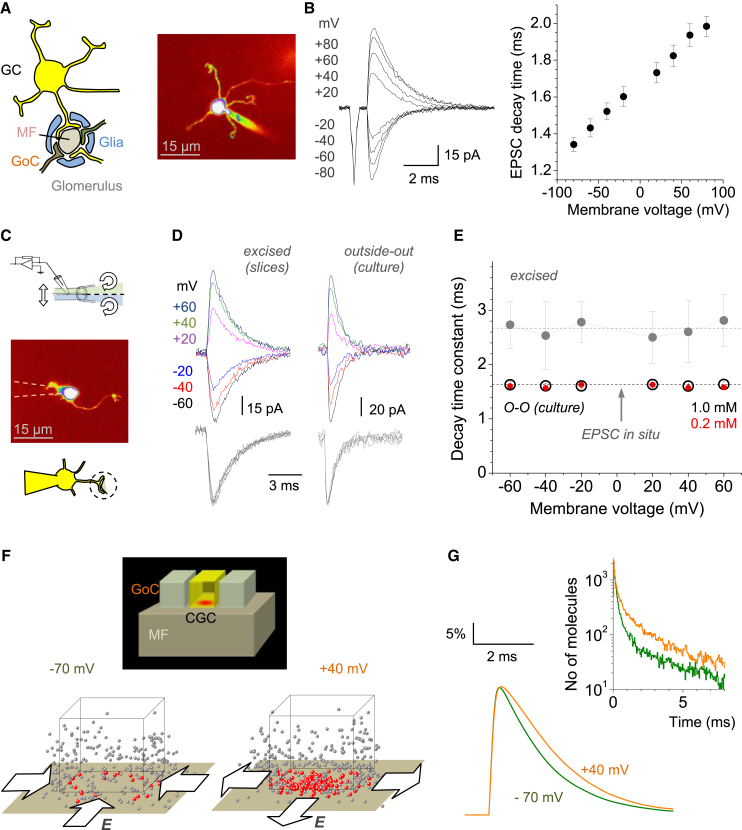
Electric Fields of Postsynaptic Currents at MF-GC Synapses Alter Diffusion of Intracleft Glutamate (A) Schematic on the left illustrates GCs receiving synapses from MFs inside the glomerulus, which also hosts Golgi cell (GoC) axons. Image on the right shows a typical GC held in whole cell, 30–100 μm deep in slice (λ_x_^2*p*^*=* 800 nm; Alexa Fluor 594 channel). (B) Traces on the left show characteristic MF-evoked EPSCs recorded in GCs at different V_m_, as indicated. The number of activated MF-GC synapses in such experiments varied from one (amplitude ∼22 pA at −70 mV) to four. Graph on the right presents statistical summary (mean ± SEM, n = 5). (C) Top view illustrates rapid application system (schematic). Middle and bottom views present a GC carefully pulled in whole-cell mode with an intact dendrite and held ∼15 μm above the slice surface (dotted lines, pipette tip out of focus); synaptic clefts are likely to remain intact during mechanical cell separation (Figure S1F). (D) Characteristic AMPAR responses to a 1 ms pulse of 1 mM glutamate recorded at different voltages (color coded); gray indicates same traces rescaled. Left and right panels show experiments in GCs excised from acute slices and in outside-out patches from cultured GCs, respectively, as indicated. (E) Summary of the AMPAR response decay time at different voltages in excised GCs (gray, mean ± SEM, n = 5; dotted line, global average) and in patches from cultured GCs in response to 1.0 mM (black, n = 5) or 0.2 mM (red, n = 5) glutamate pulse, as indicated (dotted line, EPSC decay time in situ). See Figures S1G–S1I for additional data. (F) Top view is a modeled glomerular environment; cuboids indicate fragments of GC dendrites and GoC axons (600 nm wide 50 nm apart) facing the MF axon, and red hotspot indicates glutamate release at the MF-GC synapses, in accord with Nielsen et al. (2004). Snapshots of glutamate diffusion 2 ms postrelease, at two V_m_, as indicated. For clarity, only half of simulated molecules are depicted; red and gray dots indicate molecules inside and outside the cleft, respectively, and block arrows indicate electric field direction. (G) Simulated time course of glutamate escape (inset shows number of molecules inside the cleft) and AMPAR activation (graph; scale bar, open-state occupancy) at the MF-GC synapse at −70 mV (green) and +40 mV (orange). See Experimental Procedures for modeling details.

To optimize voltage-clamp conditions, here, we focus on synapses between cerebellar mossy fibers (MFs) and granule cells (GCs), one of the smallest, electrically compact central neurons (Diwakar et al., 2009) receiving only four excitatory inputs (Figure 1A). Glutamate released at MF-GC connections activates postsynaptic AMPA and NMDA receptors (AMPARs and NMDARs) enabling high-fidelity transmission (Chadderton et al., 2004; Saviane and Silver, 2006). It has also been reported that pharmacological saturation of metabotropic glutamate receptors (mGluRs) can facilitate the NMDAR-dependent component of evoked MF-GC responses (Kinney and Slater, 1993; Rossi et al., 1996) and that long-lasting potentiation of MF-GC transmission induced by high-frequency stimuli involves both NMDARs and mGluRs (D’Angelo et al., 1999). However, the mechanism leading to the mGluR-NMDAR-dependent synaptic plasticity has remained unidentified. Metabolic actions of mGluRs have commonly been associated with relatively slow molecular cascades involving G proteins (Ferraguti et al., 2008). It has also been shown that both NMDARs and group I mGluRs are connected to the multimeric scaffolding complex at the postsynaptic density (PSD), with the mGluR linkage being mediated by Homer proteins (Tu et al., 1998). Suppressing this linkage through the expression of the immediate early gene *Homer1a* in cultured cerebellar GCs prompted group I mGluR-dependent inhibition of NMDARs (Bertaso et al., 2010), thus potentially unmasking upregulation of NMDAR activity by local mGluRs. Bioluminescence resonance energy transfer (BRET) imaging has recently revealed that a physical interaction between postsynaptic group I mGluRs and NMDARs could underlie such effects in hippocampal neurons (Moutin et al., 2012). Whether such interactions contribute to use-dependent regulation of MF-GC transmission is not known.

Intriguingly, depolarization of cerebellar GCs above zero was reported to decelerate the decay of MF-evoked AMPAR EPSCs (Cathala et al., 2005), indirectly suggesting the involvement of glutamate electrodiffusion (Sylantyev et al., 2008). However, an alternative explanation for such deceleration is the voltage-dependent kinetics of native AMPARs, a feature reported earlier in cochlear nucleus cells (Raman and Trussell, 1995) and in retina cells (Veruki et al., 2003), although not in principal hippocampal neurons (Colquhoun et al., 1992; Sylantyev et al., 2008). Here, we examine AMPAR and NMDAR activation in GCs to determine whether glutamate electrodiffusion contributes to the shaping of MF-GC responses. We combine experiments in situ, in outside-out and nucleated patches with detailed biophysical modeling to conclude that such a ubiquitous physiological event as a postsynaptic action potential (AP) can retard glutamate escape from the cleft of MF-GC synapses due to electric field effects. Rather than affecting intrasynaptic NMDARs or AMPARs, this glutamate retardation enhances activation of high-affinity group I mGluRs, which tend to occur in the periphery of excitatory cerebellar synapses (Baude et al., 1993; Luján et al., 1997; Nusser et al., 1994). In turn, activated mGluRs rapidly (millisecond scale) facilitate currents through local NMDARs. This facilitation does not involve G protein-sensitive cascades, but it is blocked when Homer1a is expressed in the postsynaptic GCs. We also examine whether, by engaging the mGluR-NMDAR interaction mechanism, the coincidence of glutamate release and postsynaptic APs at MF-GC synapses could induce long-lasting synaptic changes altering the integrate-and-spike property in the MF-GC circuitry.

## Results

### Postsynaptic Depolarization Retards Escape of Glutamate from the Synaptic Cleft

The decay constant of AMPAR EPSCs evoked in GCs by MF stimulation increased monotonically with cell depolarization (Figure 1B). The EPSC decay slowdown at positive voltages was consistent with previous observations (Cathala et al., 2005) and remained robust when voltage-sensitive glutamate transporters were blocked with 50 μM TBOA (Figures S1A and S1B available online). A subgroup of slower and smaller EPSCs representing glutamate escaping from neighboring glomerular synapses (Nielsen et al., 2004) was readily separated out in such recordings (Figure S1C). The proportion of these “spillover” EPSCs was relatively small (11.2% ± 0.7%, n = 103 cells in control conditions), and neither this proportion nor the proportion of complete release failures (4.9% ± 0.5%) was affected by cell depolarization, thus reflecting unchanged release probability (Figure S1D; in such tests, fast EPSCs could represent up to four MF-GC synapses).

To test whether the EPSC decay deceleration can be explained by the voltage dependence of AMPARs, we set out to probe AMPAR kinetics using rapid ligand application in outside-out patches (Colquhoun et al., 1992). Because AMPARs in GCs in situ are exclusively intrasynaptic and thus absent from the soma (Cathala et al., 2005; DiGregorio et al., 2002; Silver et al., 1996), we excised GCs in whole-cell mode aiming to preserve their short dendrites carrying AMPARs (Figures 1C and S1E). The success rate of these experiments was low: we documented evoked AMPAR currents only in five out of otherwise successful 112 whole-cell excisions. In all cases, however, AMPAR kinetics were clearly voltage independent (marked “excised” in Figures 1D and 1E). However, the current decay was notably slower than that of EPSCs in situ (2.63 ± 0.43 ms and 1.61 ± 0.07 ms, n = 5 and n = 23, respectively; p < 0.001; V_m_ = −70 mV). The simplest explanation for this discrepancy was that presynaptic membranes were still attached to the excised GC dendrite: indeed, intact synaptic clefts are common in electron micrographs of synaptosomes even after tissue separation in a centrifuge (Hunt et al., 1996) (Figure S1F). With the synaptic cleft intact, externally applied glutamate has to diffuse inside to reach intracleft AMPARs, which slows down its concentration transient. This explanation was fully consistent with Monte Carlo simulations mimicking this scenario (Figure S1G).

Nonetheless, it was important to probe native AMPARs on the timescale comparable with EPSCs because some AMPAR subtypes show rapid desensitization. We therefore tested membrane patches from cultured GCs (6–7 days in vitro [DIV]) that do express AMPARs in the soma (Silver et al., 1996) and therefore have no diffusion barrier for applied glutamate. In these experiments, the AMPAR current decay (1.63 ± 0.05 ms at −60 mV, n = 6) was (1) indeed similar to the EPSC decay in situ, and (2) voltage independent (Figures 1D and 1E, marked “outside-out” or “O-O”). Furthermore, decreasing the glutamate pulse concentration 5-fold (from 1.0 to 0.2 mM) in the same membrane patch reduced the AMPAR response amplitude with no effect on its kinetics (Figures 1E, S1H, and S1I), thus arguing against concomitants pertinent to partial receptor saturation.

**Figure 2:**
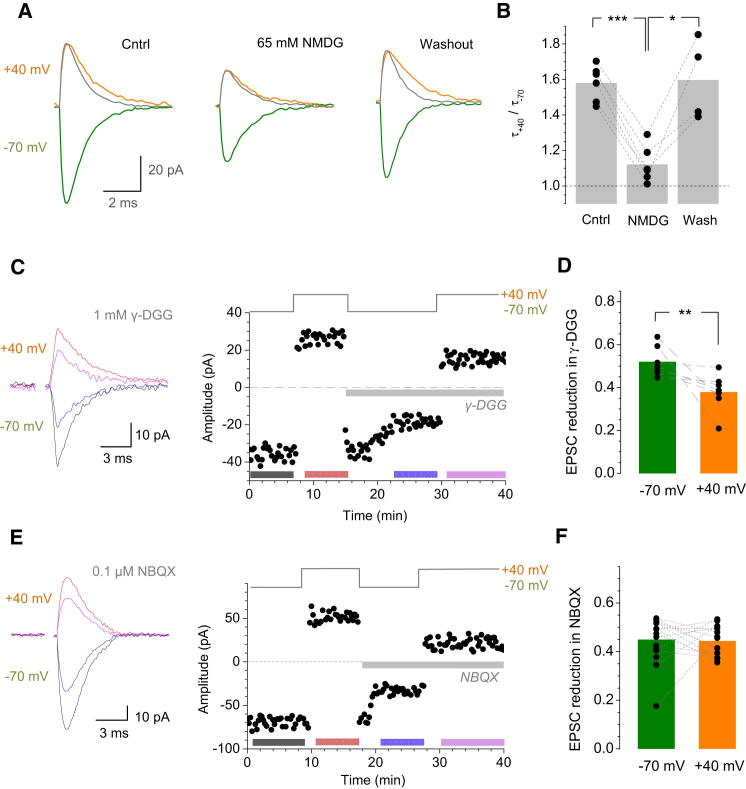
Glutamate Electrodiffusion Depends on the AMPAR Current Driving Force and Increases the Effective Intracleft Glutamate Transient upon Depolarization (A) Traces illustrate single-cell example of EPSCs recorded in control (Cntrl) bath solution (124 mM NaCl), with reduced sodium content (65 mM NMDG + 59 mM NaCl), and washout, as indicated; gray trace indicates EPSC at −70 mV with the amplitude normalized to that at +40 mV. (B) Summary of experiments shown in (A). Columns show average; dots present individual cells. ^∗∗∗^p < 0.005, ^∗^p < 0.05. Wash, washout. (C) Application of 1 mM γ-DGG has a smaller effect on AMPAR EPSCs at +40 mV compared to −70 mV. Traces present EPSCs (epoch average) before and after γ-DGG application at two voltages, as indicated. Graph illustrates the time course of the EPSC peak amplitude (38 release failures are not shown; details in Figure S1D), a single-cell example; color-coded bars indicate averaging epochs. (D) Summary of experiments shown in (C); other notations are as in (B). ^∗∗^p *<* 0.008. (E) NBQX (0.1 μM, nonsaturating concentration) has similar effects on the AMPAR EPSC peak amplitude at +40 mV and −70 mV. Other notation is as in (C); 26 release failures are not shown (details in Figure S1D). (F) Summary of experiments shown in (E); other notations are as in (D). See Figures S2B and S2C for further details.

To test whether the electric field effect on EPSCs was biophysically plausible, we integrated the environment of MF-GC synapses (Nielsen et al., 2004) into the tested Monte Carlo model that incorporates glutamate electrodiffusion in the cleft (Savtchenko and Rusakov, 2007; Sylantyev et al., 2008) (Experimental Procedures). Simulations readily reproduced the voltage asymmetry of the EPSC decay (Figures 1F and 1G), which remained robust over a physiological range of synaptic sizes and AMPAR numbers (Figure S2A). If the decay asymmetry indeed relies on intracleft electric fields, then decreasing the current at the same voltage should reduce this asymmetry. To test this, we recorded AMPAR EPSCs while halving the extracellular free sodium by partly replacing extracellular NaCl with N-methyl-D-glucamine (NMDG, 65 mM). This manipulation did indeed decrease both the amplitude and the voltage asymmetry of EPSCs (the decay constant ratio at +40 and −70 mV, τ_+40_ /τ_-70_, was reduced in NMDG from 1.59 ± 0.11 to 1.15 ± 0.05, n = 6, p < 0.005; Figures 2A and 2B).

Another prediction consequential to the electrodiffusion mechanism was that the effective concentration (or dwell time) of glutamate inside the cleft should increase upon current reversal (Sylantyev et al., 2008). To test this, we used the low-affinity AMPAR antagonist γ-DGG: its efficiency is inversely related to the intracleft glutamate concentration (Christie and Jahr, 2006; Wadiche and Jahr, 2001), in a voltage-independent manner (Sylantyev et al., 2008). We found that partial AMPAR blockade by 1 mM γ-DGG was significantly less efficient at positive holding voltages V_m_ (EPSC reduction by 52% ± 3% at −70 mV compared to 38% ± 3% at +40 mV, p *<* 0.008, n = 7, Wilcoxon paired test; Figures 2C and 2D), suggesting a greater intracleft glutamate transient at positive V_m_.

To test whether other unknown voltage-dependent conductance could explain this result, we applied a nonsaturating concentration of the high-affinity AMPAR antagonist NBQX (0.1 μM): its inhibitory effect should not depend on local glutamate concentration. In contrast to γ-DGG, the effect of NBQX on the EPSC peak amplitude was indeed voltage independent (Figures 2E and 2F). Furthermore, NBQX decelerated the EPSC decay at negative voltages while accelerating it at positive voltages (Figures S2B and S2C), thus reproducing the outcome of the NMDG experiments above (Figures 2A and 2B). Reassuringly, data from both NBQX and NMDG tests were consistent with the simulated relationship between the current amplitude and the decay asymmetry (Figure S2D).

### Postsynaptic Spikes Can Modulate Activation of Perisynaptic mGluRs by Released Glutamate

**Figure 3:**
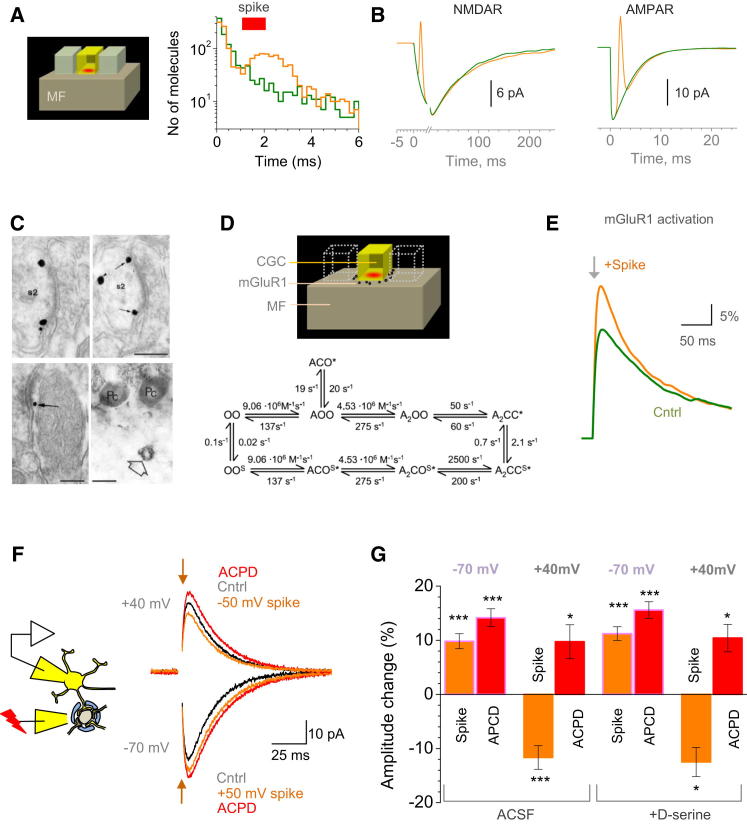
A Postsynaptic Spike Coincident with Glutamate Release Modulates Activation of Perisynaptic Group I mGluRs thus Affecting Local NMDARs (A) Inset shows model geometry (as in Figure 1F); plot presents simulated time course for the number of glutamate molecules remaining in the cleft, with (orange) and without (green) a postsynaptic AP (red bar indicates AP duration). (B) Simulated time course of AMPAR (left) and NMDAR (right) activation in baseline conditions (green) and with a coincident postsynaptic AP (orange), as indicated; combined AMPAR+NMDAR EPSC is shown in Figure S3E. (C) Published examples of pre-embedding silver-intensified immunogold labeling revealing mGluR1 at the periphery of excitatory cerebellar synapses on principal cells (top panels, adjacent sections) and interneurons (bottom left); some cerebellar glomeruli are clearly stained for mGluR1 (immunoperoxidase). Modified from Baude et al. (1993), with permission. (D) Schematic illustrating glomerular environment model as in (A) plus group I mGluR (black dots) scattered in the synaptic periphery. Kinetic diagram for mGluR1 kinetics (Marcaggi et al., 2009). (E) Traces show simulated activation time course of perisynaptic mGluR1 upon glutamate release without (green) and with a coincident AP (orange), as indicated. (F) Schematic (left) and characteristic traces (right) showing the effect of mGluR saturation (200 μM ACPD) and of the spike-release pairing on NMDAR EPSCs at two V_m_, as indicated: one-cell example. The spike-only traces were subtracted from the spike-release pairing traces to remove the pulse (Figure S3F). (G) Summary of experiments shown in (F). Columns and error bars present change (mean ± SEM) in the NMDAR EPSC amplitude relative to baseline in response to a spike-release pairing or ACPD application, as indicated; experiments in baseline conditions (ACSF, n = 6) and with 1 mM D-serine in the bath (n = 4), as indicated. ^∗∗∗^p < 0.005; ^∗^p < 0.05.

Although the aforementioned tests detect glutamate electrodiffusion per se, they rely on sustained cell depolarization above zero, which is an unlikely physiological scenario. We therefore asked if a single postsynaptic AP, by briefly reversing the synaptic current, could influence glutamate diffusion and thus receptor activation in the cleft. Simulations did indicate that an AP can retard escape of released glutamate, briefly increasing its concentration (tail) transient 3- to 4-fold (Figure 3A). At the same time, EPSCs perturb the intracleft levels of pre-equilibrated Na^+^, K^+^, and Cl^−^ by 15%–20% (Figures S3A–S3C); although incorporated in the model, this perturbation per se has little effect on synaptic currents, reflecting relative saturation of receptors by these ions (Figures S3A, S3B, and S3D). However, the model predicted no detectable effect of the AP-evoked glutamate retardation on local AMPARs or NMDARs (Figures 3B and S3E). Our subsequent experiments supported this prediction; see sections below.

Glutamatergic signaling at MF-GC synapses extends, however, beyond AMPARs or NMDARs. Activation of mGluRs boosts transmission at these synapses (Kinney and Slater, 1993; Rossi et al., 1996), and mGluR1s are commonly found at the postsynaptic periphery of excitatory connections in the cerebellum (Baude et al., 1993; Luján et al., 1997; Nusser et al., 1994) (Figure 3C). We asked therefore whether the AP-dependent changes in glutamate escape could affect local mGluR1s: high-affinity receptors outside the cleft could be particularly sensitive to glutamate retardation (Min et al., 1998). First, we tested the theoretical plausibility of such effects, by incorporating perisynaptic mGluR1s into the MF-GC synapse model (Figure 3D), with the mGluR1 kinetics adapted from a FRET study of induced conformational changes in mGluR1s (Marcaggi et al., 2009). Our simulations readily predicted that an AP generated during or immediately after MF glutamate release could robustly increase activation of perisynaptic mGluR1s (Figure 3E).

### Modulation of NMDARs by Group I mGluRs and by Spikes Coincident with Release

If glutamate retardation indeed boosts mGluR1 activation, we should be able to detect this as an enhancement of NMDAR currents at MF-GC synapses (Kinney and Slater, 1993; Rossi et al., 1996). Indeed, the wide-range mGluR agonist ACPD (200 μM) boosted NMDAR EPSCs, at both positive and negative V_m_ (by 14% ± 2% and 10% ± 3%; p < 0.005 and p < 0.05, respectively; n = 6; Figures 3F and 3G; isolated NMDAR currents were routinely recorded in zero Mg^2+^). We next tested if pairing a release event with a brief voltage-reversing spike has any influence on NMDAR activation. In these experiments, a 2 ms pulse was applied 0.5 ms before the MF stimulus; the spike-only trace was routinely subtracted from the pairing trace providing the resulting trace with no pulse artifacts (Figure S3F). In contrast to the facilitatory action of ACPD at positive and negative V_m_, pairing enhanced NMDAR EPSCs at negative while reducing it at positive V_m_ (by 10% ± 1% and by 9% ± 2%, respectively; n = 6; p < 0.005; Figures 3F and 3G). These effects were not a contaminant action of an mGluR agonist on the NMDAR coagonist site (Contractor et al., 1998) because saturating the coagonist site with 1 mM D-serine did not change the outcome (Figure 3G).

**Figure 4:**
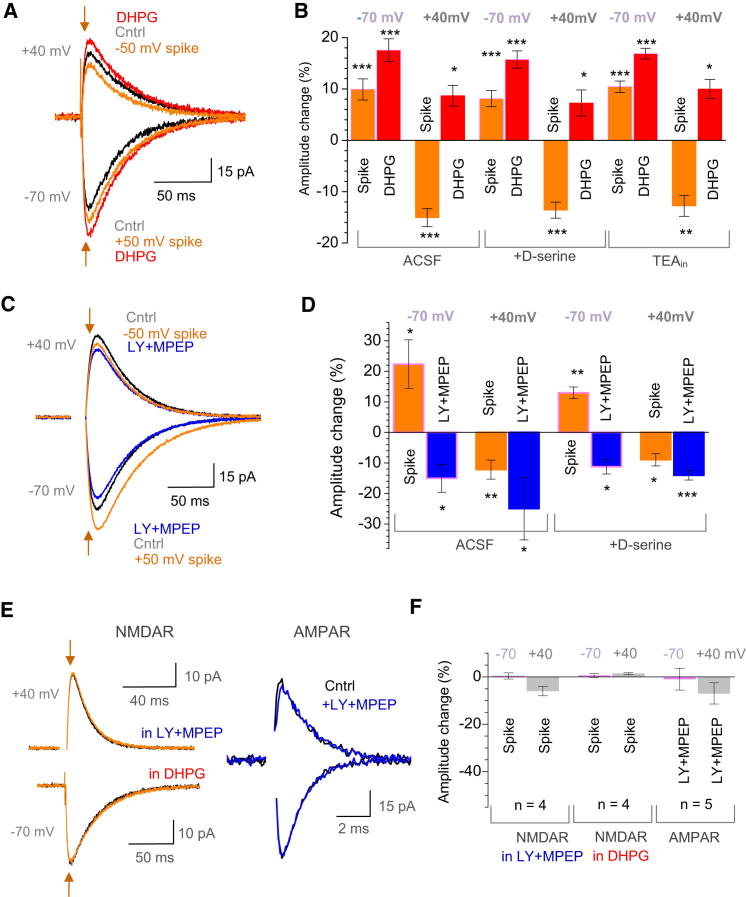
Modulation of NMDAR EPSCs by the Coincident Postsynaptic Spike Depends on Group I mGluRs (A) The effect of mGluR saturation (100 μM DHPG) and of the spike-release pairing on NMDAR EPSCs at two V_m_, as indicated: one-cell example. The spike-only traces were subtracted from the spike-release pairing traces to remove the pulse (Figure S3F). (B) Summary of experiments shown in (A). Columns and error bars present average change (mean ± SEM) in the NMDAR EPSC amplitude relative to baseline in response to spike-release pairing or DHPG application, as indicated; control conditions (normal ACSF, n = 6), with 1 mM extracellular D-serine (n = 4), and with 1 mM intracellular TEA (K-gluconate-based intracellular solution, n = 4), as indicated. ^∗∗∗^p < 0.005; ^∗∗^p < 0.01; ^∗^p < 0.05. (C) The effect of mGluR blockade (100 μM LY + 200 nM MPEP) and of the spike-release pairing on NMDAR EPSCs at two V_m_, as indicated: one-cell example. Other notations are as in (A). (D) Summary of experiments shown in (C). Columns and error bars present average amplitude change (mean ± SEM) during spike-release pairing or LY+MPEP application, as indicated; control conditions (normal ACSF, n = 6), and with 1 mM extracellular D-serine (n = 4), as indicated, and other notations are as in (B). (E) Example traces of NMDAR EPSCs, with (orange) and without (black) spike-release pairing under mGluR1 blockade with LY+MPEP or saturation with DHPG, as indicated; example traces of AMPAR EPSCs in control (black) and under mGluR blockade (blue); V_m_ shown. (F) Summary of experiments shown in (E). Columns and error bars present average change in the peak EPSC amplitude (mean ± SEM), as indicated. See Figure S4 for the time course analyses of mGluR-dependent NMDAR actions and for further data on voltage-independence of mGluR1 ligands.

Qualitatively identical results were obtained using group I mGluR agonist DHPG, with or without D-serine, and also with intracellular TEA (1 mM) loaded to suppress potassium conductance (Figures 4A and 4B). Conversely, blockade of group I mGluRs with specific antagonists LY367385 (LY, 100 μM) and MPEP (200 nM, applied together) robustly reduced the amplitude of NMDAR EPSCs, at both negative and positive V_m_ (Figures 4C and 4D; by 15% ± 5% and 25% ± 10%, respectively, n = 5; p < 0.05), with or without D-serine. Again, in contrast to the voltage-independent inhibitory actions of LY+MPEP, voltage-reversing spikes had opposite effects on NMDAR EPSC peak amplitudes at negative versus positive V_m_ (Figures 4C and 4D). These phenomena were no less robust when the effect of LY+MPEP and spikes was gauged using the net difference between control and test EPSC traces, rather than the EPSC peak amplitude value (Figures S4A–S4D). Similar results were obtained using the wide-spectrum mGluR antagonist S-MCPG (200 μM) (Figures S4E and S4F), suggesting that mGluR subtypes other than group I do not add appreciably to the effect.

The aforementioned observations indicated that during glutamate release, local group I mGluRs were neither saturated nor completely silent and that the effect of pharmacological mGluR saturation or blockade on NMDAR currents was voltage independent. We further confirmed that the latter was the case across the range of physiological voltages (Figure S4G). Finally, we asked whether NMDARs and mGluRs were both essential for the underlying mechanism. Blockade or saturation of group I mGluRs abolished any effects of spike-release pairing on NMDAR EPSCs (Figures 4E and 4F). Similarly, pharmacologically isolated AMPAR EPSCs were insensitive to spike-release pairing (Figures 4E and 4F); in these experiments, the NMDAR blocker APV had no effect on a stimulus deflection at either voltage (Figure S5A).

### GC mGluR1s Modulate Local NMDARs on a Millisecond Timescale

**Figure 5:**
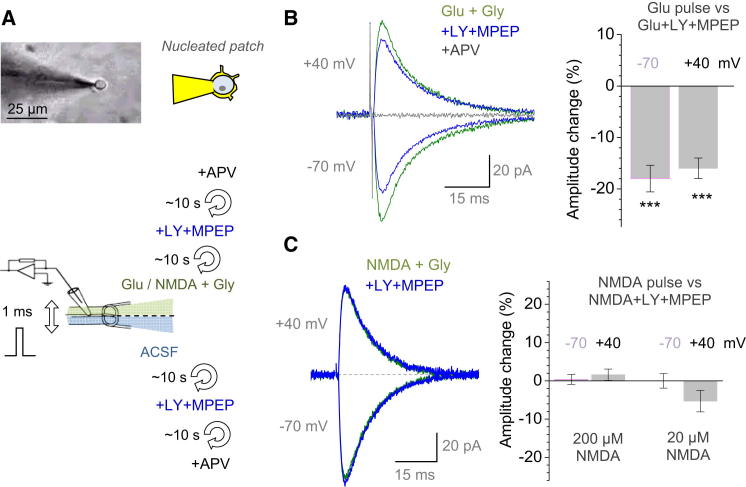
Spike-Dependent Activation of Group I mGluRs Boosts NMDAR Currents on the Millisecond Scale (A) Illustration and schematic of nucleated-patch experiment in acute slices. Upper panels show patch configuration (held 100–150 μm above the slice surface); lower panel is a schematic of fast-application fast-exchange solution experiment in which both θ-glass channel solutions are replaced within ∼10 s, as indicated. (B) Isolated NMDAR currents (AMPARs and GABARs are blocked) evoked in nucleated patches by 1 ms pulses of 1 mM glutamate are inhibited by group I mGluR blockade at both negative and positive V_m_; traces, characteristic one-cell example. Bar graphs illustrate average change (±SEM, n = 5). ^∗∗∗^p < 0.005. (C) Same protocol as in (B) but with 200 μM NMDA pulses (n = 5; traces: one-cell example) and 20 μM NMDA pulses (n = 4), as indicated. Bar graph illustrates statistical summary indicating no detectable effect of the mGluR1 blockade throughout tests; notation is as in (B). See Figure S5C for further details and example traces.

For NMDAR EPSCs to be affected by mGluRs shortly after release of glutamate, the mGluR-NMDAR interaction has to be rapid. We examined its timescale using fast ligand application (<1 ms resolution) in nucleated patches of GCs (Experimental Procedures; Figure 1C). This experimental configuration leaves the small GC soma virtually intact (Figures 5A and S5B), thus helping to preserve the cellular machinery of membrane proteins while avoiding any presynaptic or network influences.

A 1 ms pulse of 1 mM glutamate (+1 mM glycine) evoked a robust NMDAR current in the nucleated patch (Figure 5B), which was comparable with NMDAR EPSCs in situ. However, in the same patch, the NMDAR response to the same pulse, but in the presence of LY+MPEP (solution exchange in both θ-glass barrels took ∼10 s), was significantly smaller, at both negative and positive V_m_ (by 18% ± 3% and 16% ± 2%, respectively, n = 5; p < 0.005; Figure 5B). Thus, a 1 ms exposure of group I mGluRs to glutamate was sufficient to boost NMDAR currents. This effect was not due to a contaminant action of LY+MPEP and not because of the constituent activity of mGluRs because the same experiment with NMDA applied instead of glutamate showed no effect of LY+MPEP on NMDAR responses (amplitude change 0.0% ± 1.3% at −70 mV and 1.7% ± 1.5% at +40 mV, n = 5; p > 0.8; Figure 5C). The result was the same when the NMDA pulse was ten times lower (20 μM; Figures 5C and S5C), thus arguing against any concomitant effects of partial NMDAR saturation. We also confirmed that isolated activation of mGluRs (with ACPD or DHPG) in the same NMDAR-containing nucleated patch evoked no detectable response (Figure S5C). Further evidence for a millisecond-range interaction between group I mGluRs and local NMDARs was obtained in experiments described below.

### Rapid Modulation of Postsynaptic NMDARs by Group I mGluRs Does Not Require Ca^2+^ Signaling but Involves Homer-Containing Scaffold

**Figure 6:**
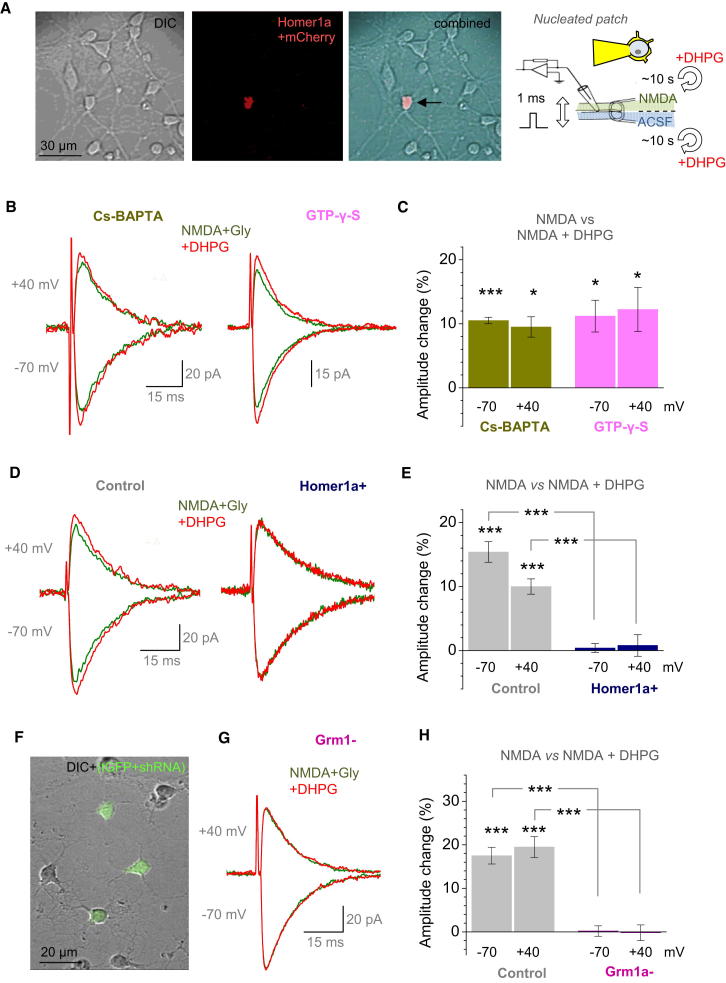
Group I mGluRs Rapidly Boost Activation of Local NMDARs in GCs through a Homer1a-Dependent Molecular Linkage (A) An example of GC cultures (left image, DIC), with a proportion of cells successfully cotransfected with mCherry and Homer1a (middle image is an mCherry-positive cell, λ_x_^2P^ = 890 nm, fluorescence channel; right image is merged). Right panel is a schematic of experiments using fast ligand application in nucleated patches, as indicated. (B) Characteristic one-cell examples. NMDAR currents evoked in nucleated patches by 1 ms pulses of 200 μM NMDA are boosted by group I mGluR saturation with DHPG, at both negative and positive V_m_, in the presence of Cs-BAPTA (40 mM, left) or GTP-γ-S (500 μM, right) in the pipette. (C) Statistical summary of experiments depicted in (B). Columns and error bars present average change (mean ± SEM) for BAPTA tests (n = 3) and in the presence of GTP-γ-S (n = 4), as indicated. ^∗∗∗^p < 0.005; ^∗^p < 0.05. (D) One-cell examples. NMDAR currents evoked by 1 ms pulses of 0.2 mM NMDA are boosted by DHPG in wild-type (left) but not in Homer1a expressing cells (right), as indicated; scale bars apply to both examples. (E) Summary of tests depicted in (D) for nontransfected (control, n = 5) and Homer1a+ (n = 5) cells. Other notations are as in (C). (F) An example of GC cultures (DIC image) with a proportion of cells successfully cotransfected with a lentiviral vector coding TurboGFP and shRNA against mGluR1 (green fluorescence). See Figure S6E for mGluR1 immunostaining control. (G) One-cell example. In transduced cells (Grm1-, suppressed expression of mGluR1s), NMDAR currents evoked by 1 ms pulses of 0.2 mM NMDA are insensitive to DHPG. (H) Statistical summary of tests shown in (G) for nontransduced (or transduced with nonsilencing lentiviral vector; control, n = 5) and Grm1- (n = 9) cells. Other notations are as in (C). See Figure S6 for pertussis toxin data, amplitude versus effect controls for Homer1a experiments, and immunostaining control of mGluR1 gene silencing.

To test the hypothesis that the rapid group I mGluR-NMDAR interaction involves Homer proteins (Bertaso et al., 2010; Moutin et al., 2012), we probed nucleated patches of cultured GCs that were cotransfected with Homer1a and, for identification, with mCherry under *Synapsin* promoter (Figure 6A; Experimental Procedures). Again, we used a system that provides ∼1 ms ligand applications and a full exchange of solutions within ∼10 s, thus enabling highly sensitive pharmacological protocols in the same nucleated patch.

First, we found that in wild-type cells, adding intracellular Cs-BAPTA (40 mM) to suppress intracellular Ca^2+^ transients failed to abolish the facilitatory action of DHPG (NMDAR responses increased by 10% ± 1% and 9% ± 2% at −70 mV and +40 mV, respectively, n = 3, p < 0.001 and p < 0.05; Figures 6B and 6C). Second, saturating the activity of membrane-bound G protein-coupled receptors with GTP-γ-S (500 μM) in the excised soma had no effect on this facilitation either (DHGP-dependent increase: 11% ± 3% and 12% ± 4%, at −70 mV and +40 mV, respectively, n = 4; p < 0.05; Figures 6B and 6C). As expected, pertussis toxin cell loading yielded a similar result (Figure S6A). These observations thus argued against the involvement of the classical G protein cascades.

In contrast, in Homer1a-transfected cells, DHPG had no effect on NMDAR currents (change 0.5% ± 0.9% and 0.7% ± 1.8% at −70 mV and +40 mV, respectively, n = 5; p > 0.62 at least), whereas in nontransfected cells from the same cultures, DHPG robustly facilitated NMDAR responses (by 15% ± 2% and 9% ± 1% at −70 mV and +40 mV, respectively, n = 5; p < 0.001; Figures 6D and 6E). The latter effect was fully consistent with our observations in situ (Figures 4A and 4B), and it could not be explained by a systematic difference in the NMDAR current amplitude between transfected and nontransfected cells (Figures S6C and S6D). To further confirm the molecular identity of mGluRs involved and to rule out nonspecific effects of mGluR ligands (Contractor et al., 1998), we silenced the mGluR1 gene (*Grm1*) using a shRNA approach, with a scrambled sequence in nonsilencing lentiviral vector for control, and TurboGFP expression in lentivirus-infected cells (Experimental Procedures; Figures 6F and S6E). Again, the facilitatory effect of DHPG on NMDARs was fully suppressed in transduced cells (difference 0.2% ± 1.2% and −0.2% ± 1.8% at −70 and +40 mV, respectively, n = 9), whereas in nontransduced (or transduced with control, nonsilencing vector) cells, it was as prominent as in situ (18% ± 2% and 19% ± 2% at −70 and +40 mV, respectively; both at p < 0.005, n = 5; Figures 6G and 6H). Thus, the Homer-dependent linkage between mGluR1 and the NMDAR-associated PSD scaffold is the likely mechanism underlying rapid interaction between the two receptors, as documented here.

### Release-Spike Coincidence Triggers Lasting, mGluR- and NMDAR-Dependent Changes in Signal Integration Properties of the MF-GC Circuitry

**Figure 7:**
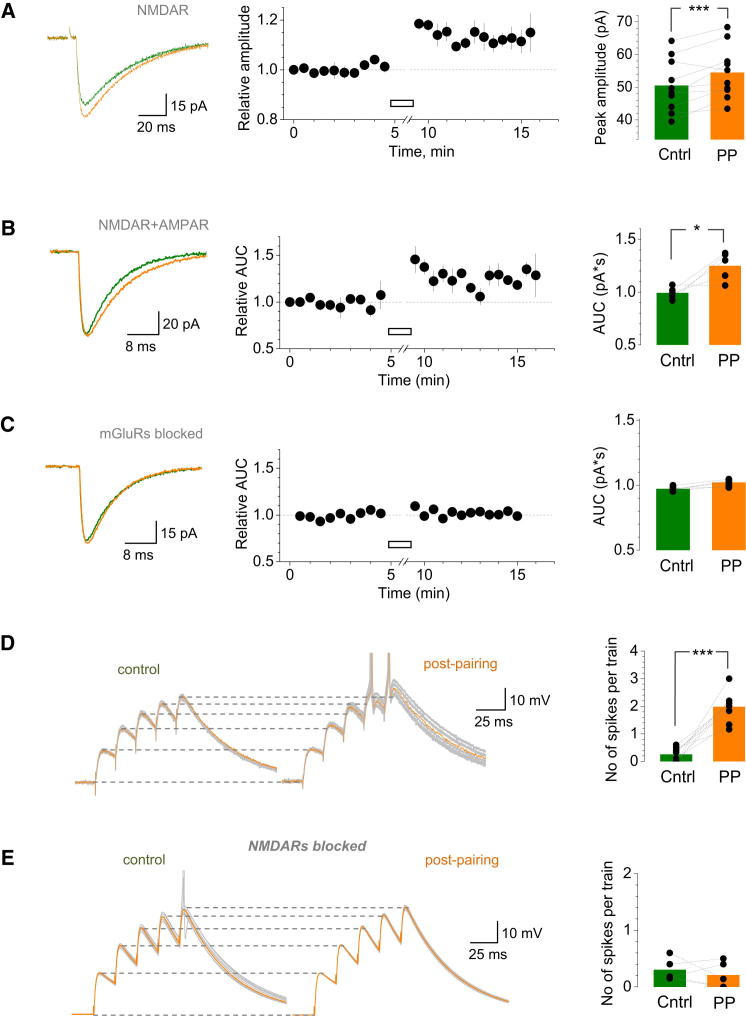
Pairing Evoked Glutamate Release with a Postsynaptic Spike Induces a Lasting Enhancement of Basal Transmission at MF-GC Synapses (A) Traces illustrate average NMDAR-mediated EPSCs before (green) and after (orange) 20 episodes of pairing (one-cell example); AMPAR, GABA_A_Rs, and GABA_B_Rs are blocked. Plot shows average time course of the NMDAR EPSC peak amplitude (n = 10); open bar presents pairing epoch. Bar graph presents summary, dots indicate individual experiments, and column shows average peak amplitudes. ^∗∗∗^p *<* 0.001. (B) Traces illustrate characteristic NMDAR- and AMPAR-mediated EPSCs before (green) and after (orange) pairing (one-cell example). Time course and bar graphs depict EPSC charge transfer values (AUC; n = 5); GABA_A_ and GABA_B_ receptors are blocked. Other notations are as in (A). ^∗^p *<* 0.035. (C) Same experiments as in (B) but with the pharmacological blockade of mGluRs (200 μM S-MCPG, n = 5). Other notations are as in (A) and (B). (D) Traces illustrate characteristic AMPAR- and NMDAR-dependent EPSPs (current clamp) evoked in a GC by five MF stimuli before and after spike-release pairing (protocol as above, spikes are truncated), as indicated. Green and orange lines depict an individual trace for comparison. Gray lines show four to six consecutive traces. Dotted lines depict summation of consecutive responses before and after pairing. Bar graph presents statistical summary: the average number of spikes per response before and 3–4 min after pairing, as indicated; dots indicate individual experiments (n = 9). ^∗∗∗^p < 0.005. (E) Experiments as in (D) but with NMDARs blocked using 100 μM D-APV. Other notations are as in (D). Average number of spikes per train before and 3–4 min after pairing is 0.30 ± 0.09 and 0.21 ± 0.10, respectively (n = 5). p > 0.8. See Figure S7A for mGluR1 blockade control and Figures S7B and S7C for NEURON model simulations of postsynaptic events in GCs.

Our results have thus suggested that the temporal coincidence of the postsynaptic spike and glutamate release at MF-GC synapses enhances activation of perisynaptic (intraglomerular) mGluR1s in GCs. Does this coincidence have any long-term consequences? We found that the peak amplitude of NMDAR EPSCs was increased for at least 5–10 min after 20 episodes of spike-release pairing by 11% ± 2% (n = 10; p *<* 0.001; Figure 7A). When both AMPARs and NMDARs were left unblocked, the pairing protocol produced a long-term increase of the EPSC charge transfer, or the area under the curve (AUC), by 25% ± 15% (n = 5; p < 0.035; Figure 7B). The latter increase was consistent with the potentiation of the slower (and smaller) NMDAR-dependent, as opposed to the faster AMPAR-dependent, EPSC component. Conversely, spike-release pairing produced no lasting changes when group I mGluRs were blocked (n = 5; Figure 7C).

Because GCs could operate in vivo at high frequencies (Chadderton et al., 2004; Rancz et al., 2007; Saviane and Silver, 2006), this coincidence-dependent plasticity could have important consequences for input integration during short presynaptic bursts. To test this, we compared summation of EPSPs (current clamp) during trains of five stimuli, before and after spike-release pairing. In cerebellar GCs, EPSPs are much slower and more NMDAR dependent than EPSCs (Chadderton et al., 2004; D’Angelo et al., 1995). The interstimulus interval was adjusted (around 20 ms) so that in baseline conditions, the burst induced, on average, between none and one postsynaptic AP. For spike-release pairing, we used ∼2 ms pulses just above the GC threshold, to ensure that the ensuing AP was close to its native waveform. We found that, following 20 episodes of singe-pulse pairing, the summated response to the same train of stimuli was substantially larger, with the occurrence of postsynaptic spikes being increased many fold (1.98 ± 0.18 and 0.25 ± 0.08 spikes per train, respectively, n = 9; p < 0.001; Figure 7D). Consistent with single-stimulus-evoked EPSC data (Figure 7B, traces), the enhanced summation could be fully explained by the decelerated decay of EPSPs postpairing (due to an increased contribution of the slower NMDAR-dependent component). The pairing-induced potentiation was abolished when either NMDARs or mGluR1s were blocked (Figures 7E and S7A, respectively).

Finally, to test if the observed changes in the integrate-and-spike properties were consistent with cell biophysics, we explored a well-tested NEURON model of the GC (http://senselab.med.yale.edu; model = 116835) (Diwakar et al., 2009). First, simulations confirmed that the AP waveform varies very little across the compartments of this electrically compact cell (Figure S7B). Second, we could readily reproduce the experimental relationship between the prolonged EPSP decay and synaptic integration by mimicking the pairing-induced change of the synaptic current kinetics (Figure S7C).

## Discussion

The findings of this study are several fold. First, we have found that AMPAR EPSCs evoked in electrically compact cerebellar GCs by stimulation of MFs decay slower upon cell depolarization. This cannot be explained by the V_m_ sensitivity of AMPARs because the kinetics of AMPAR responses in excised patches of GCs to brief pulses of glutamate were voltage independent. The blockade of voltage-dependent glutamate transporters had no effect on the voltage asymmetry of the EPSC decay either, whereas reducing the current driving force (at the same V_m_) did reduce it. The fast-dissociating AMPAR antagonist γ-DGG was less efficient at positive holding voltages, suggesting that EPSC reversal increases the effective concentration of glutamate released into the synaptic cleft. These observations coupled with Monte Carlo simulations have suggested that in the MF-GC circuitry, synaptic currents influence escape of charged glutamate from the cleft, the phenomenon first detected in hippocampal CA3-CA1 synapses (Sylantyev et al., 2008).

To understand whether the interaction between synaptic currents and intracleft glutamate at MF-GC synapses had an adaptive physiological role, we asked if a common physiological event, the postsynaptic AP, could have a significant effect on glutamate diffusion in the cleft. Our biophysical model did predict that postsynaptic spikes should briefly yet significantly decelerate escape of released glutamate, but it also predicted little consequences for intracleft AMPARs or NMDARs. However, excitatory cerebellar synapses often express high-affinity group I mGluRs at the postsynaptic periphery, and our model predicted that these receptors might be affected by AP-driven changes in glutamate escape. Indeed, retarded diffusion was shown previously to enhance activation of perisynaptic (axonal) mGluRs in hippocampal MFs (Min et al., 1998). Here, we have found that the spike-release pairing boosts activation of perisynaptic group I mGluRs, which is reflected in an increased activation of local NMDARs (but not AMPARs). Notably, the effect was opposite when the signs of the EPSC and the coincident voltage-reversing spike were reversed. Although it would be difficult to fully exclude any yet unknown contributors to this phenomenon, the result strongly implicated electric field effects: deceleration of glutamate escape by a depolarizing spike at negative V_m_ should boost activation of mGluRs (and therefore NMDARs), whereas acceleration of glutamate escape by a hyperpolarizing spike at positive V_m_ should decrease it. In contrast, the effects of saturation or blockade of mGluRs on NMDAR currents were voltage independent.

The mechanism underlying interaction between mGluRs and NMDARs has traditionally been thought to involve the ubiquitous, relatively slow PKC-/PKA- and IP_3_-dependent metabotropic cascades or, alternatively, tyrosine kinase signaling involving Pyk2 kinase and the *src* family kinases Src and Fyn (Ferraguti et al., 2008). However, our data in situ implied that this interaction should occur on the timescale of synaptic responses. We used fast application of receptor ligands to nucleated patches of GCs and found that, indeed, activation of group I mGluRs could alter NMDAR kinetics within less than 1 ms. Furthermore, buffering postsynaptic Ca^2+^ with Cs-BAPTA or blocking important G protein interactions in the postsynaptic cell had no effect on the rapid mGluR-NMDAR interaction. Although Ca^2+^ buffering cannot fully suppress Ca^2+^ signaling on the nanoscale, these results suggested that the underlying mechanism may involve a physical link between the two receptors.

In fact, studies of the protein PSD scaffolds have long documented such a link. It has been shown that the Shank proteins (Shank1, Shank2, and Shank3) form a large multimeric complex at the PSD base connecting to group I mGluRs and to NMDARs through the dimeric adaptor proteins, Homer (Homer1b, Homer1c, Homer2, and Homer3, all containing an important coiled-coil domain involved in molecular linkage) and the GKAP-PSD95 protein complex, respectively (Tu et al., 1998). A critical role of Shank1B-Homer1b interactions in relating activation of group I mGluRs to postsynaptic Ca^2+^-dependent signaling has been shown in cultured hippocampal neurons (Sala et al., 2005). Importantly, the interaction between constitutively expressed coiled-coil-containing Homer and group I mGluRs could be antagonized by the protein product of an immediate early gene *Homer1a* induced by intense neural activity (Xiao et al., 1998). The Homer1a protein does not contain the coiled-coil domain and thus acts as a dominant-negative monomeric regulator of the respective protein-protein assembly, thus potentially interrupting the molecular link between mGluR1a and NMDARs. Expression of Homer1a protein in the brain is uniquely dynamic: when induced by the maximum electroconvulsive seizure, it shows significant presence in several areas, including the cortex, hippocampus, and cerebellum (Xiao et al., 1998). At the synaptic level, constitutive Homer1b protein has been found at PSDs of excitatory cerebellar synapses (Xiao et al., 1998), consistent with the scenario of Homer1a actions in our experiments. In cultured GCs, expression of Homer1a inhibited NMDAR currents during mGluR coactivation (Bertaso et al., 2010). Importantly, a Homer1a-dependent physical link between postsynaptic group I mGluR (mGluR5a) and NMDARs has recently been revealed using single-cell BRET imaging in hippocampal neurons (Moutin et al., 2012), thus arguing for the plausibility of rapid receptor interaction documented here. However, the underlying molecular mechanism remains to be ascertained. The two earlier studies report that expression of Homer1a enables inhibition of NMDARs by slow or sustained activation of group I mGluRs (Bertaso et al., 2010; Moutin et al., 2012), whereas our results and previous physiological observations (Kinney and Slater, 1993; Rossi et al., 1996) document enhancement of NMDARs during millisecond-scale (either synaptic or exogenous) glutamate actions on mGluRs. Although both sets of observations suggest that mGluRs use the Homer-dependent linkage to boost NMDAR activation, the dynamic range of this “gain-of-function” mechanism seems to depend on the timescale of receptor activation by glutamate.

Finally, we have shown that the coincidence of postsynaptic spikes with glutamate release is sufficient to trigger a lasting enhancement of MF-GC transmission. This mGluR- and NMDAR-dependent potentiation is reflected in a prolonged EPSP decay and alters the basic integration rule for synaptic inputs converging on GCs. In effect, it sharply increases the probability of postsynaptic spiking in response to the same burst of presynaptic APs. Although our results unveil a basic biophysical mechanism that triggers such phenomena, a separate study will be required to understand the cellular machinery of potentiation. Furthermore, it remains to be seen whether other high-affinity glutamate receptors occurring outside the cleft of MF-GC synapses, such as a proportion of NR2B-containing NMDARs (Mitchell and Silver, 2000), are also sensitive to spike-dependent glutamate escape.

The phenomenon of AP-driven glutamate escape regulation could be, in principle, relevant to other excitatory synapses in which high-affinity perisynaptic receptors play a role in the induction of plasticity, such as synapses in the barrel cortex (Egger et al., 1999) or the somatosensory cortex (Nevian and Sakmann, 2006). The extent of such phenomena would depend on the features of synaptic environment, with greater field effects arising in larger synaptic clefts, as suggested by our Monte Carlo simulations (Figure S2A). However, in cholinergic synapses, intracleft electric fields could have diametrically opposite effects compared to those described here because acetylcholine is positively charged. In contrast, GABA is a zwitterion and therefore should not be sensitive to local fields (Sylantyev et al., 2008). It is also an open question whether electric fields of synaptic currents could affect mobility and clustering of intracleft synaptic receptors carrying electric charges (Poo, 1985; Triller and Choquet, 2008). Because both APs and glutamate transients in the cleft last only 1–2 ms, their co-occurrence has to be a tightly controlled event. Indeed, it seems reasonable to argue that in high-bandwidth brain circuits such as MF-GC connections (Rancz et al., 2007), this strict coincidence requirement could reflect the need for exceptional temporal precision when triggering a homeostatic change.

## Experimental Procedures

See Supplemental Experimental Procedures for full details including abbreviations.

### Electrophysiology: In Situ

All animal experiments in this study were carried out in full compliance with the corresponding EU regulations. Parasagittal slices (250 μm) were cut from the cerebellar vermis of 25- to 30-day-old Sprague-Dawley rats and incubated for 1 hr in a solution containing 124 mM NaCl, 3 mM KCl, 1 mM CaCl_2_, 3 mM MgCl_2_, 26 mM NaHCO_3_, 1.25 mM NaH_2_PO_4_, 10 mM D-glucose, bubbled with 95:5 O_2_/CO_2_ (pH 7.4). In the recording chamber, the external solution also contained 2 mM CaCl_2_ and 2 mM MgCl_2_ for AMPAR EPSC recording, and 2 mM CaCl_2_ + zero Mg^2+^ for NMDAR EPSC recording. In addition to 1 μM CGP-55845 and 100 μM picrotoxin, AMPAR and NMDAR responses were isolated by adding 100 μM D-APV and 10 μM NBQX, respectively. Where required, group I mGluRs were blocked with 100 μM LY and 200 nM MPEP applied together (or 200 μM S-MCPG where indicated). The pipette solution for voltage-clamp recordings contained 117.5 mM Cs-gluconate, 17.5 mM CsCl, 10 mM KOH-HEPES, 10 mM BAPTA, 8 mM NaCl, 5 mM QX-314, 2 mM Mg-ATP, 0.3 mM GTP; for current clamp, it contained 126 mM K-gluconate, 4 mM NaCl, 5 mM HEPES, 15 mM glucose, 1 mM MgSO_4_ × 7H_2_O, 2 mM BAPTA, 3 mM Mg-ATP (pH 7.2, 295 mOsm in both cases; pipette resistance 7–9 MOhm). Recordings were performed at 33°C–35°C; signals digitized at 10 kHz. MFs were stimulated with a bipolar tungsten electrode placed in the white matter near the gyrus crest (Garthwaite and Batchelor, 1996). Smaller and slower “spillover” AMPAR EPSCs and release failures (Figures S1C and S1D) were excluded from consideration.

### Electrophysiology: Rapid Ligand Application in Outside-Out and Nucleated Patches

Patches were excised from GCs held in whole-cell mode (Figures S1E and S5B). We adapted the fast-application method from Colquhoun et al. (1992) using a θ-glass application pipette pulled out to an ∼200 μm tip diameter, as described earlier (Sylantyev et al., 2008). Three microcapillaries inserted into each θ-glass channel enabled solution replacement within ∼10 s; pressure was adjusted using the two-channel PDES-02DX pneumatic microejector (npi electronic GmbH) using compressed nitrogen. Electric pulses were applied via a constant-voltage stimulus isolator and adjusted using a water test (Sylantyev et al., 2008). Patches were held 100–150 μm above the slice surface, with 4–5 mm of the θ-glass pipette tip submerged in the perfusion chamber at 33°C–35°C. We routinely checked the application temperature by placing a microthermocouple (tip diameter ∼100 μm, precision ±1°C) in the double-barrel streams, near the future position of the patch. The characteristic time constant of the rapid switch response in these settings was 150–250 μs (Sylantyev et al., 2008).

### Cell Cultures

Primary dissociated GC cultures were prepared using tissue from rat pups at P6, in line with earlier studies by Silver et al. (1996). Cells were plated on coverslips coated with poly-L-lysine and cultured in Basal Medium Eagle supplemented with 10% FBS, 25 mM KCl, 2 mM glutamine, 100 U/ml penicillin, and 0.1 mg/ml streptomycin. The cultures were maintained in a humidified incubator in 5% CO_2_ at 37°C. To restrict glial cell growth, 10 μM cytosine-β-d-arabinofuranoside was added to the cultures 24 hr after plating. The cultures were used for experiments at 6–7 DIV.

### Cell Cultures: Transfection with Homer1a and Knocking Down the mGluR1 Gene

Cultures were transfected at 5 DIV with pRK5-Homer1a (kindly provided by Julie Perroy and Laurent Fagni) using Effecten reagent (QIAGEN). A plasmid carrying mCherry under *Synapsin* promoter was a fluorescent transfection marker. mCherry and Homer1a were cotransfected at 1:2 ratio. Two days after transfection, whole-cell test recordings were made in mCherry-positive cells, with a control group from mCherry-negative cells. Data were collected from at least three different cultures.

To silence the mGlur1 gene, we have used Thermo Scientific Open Biosystems Human GIPZ Lentiviral shRNAmir library (institutional subscription). GIPZ is a miR30-based vector that drives expression of hairpin RNA and TurboGFP from the same RNA polymerase II promoter (CMV promoter); thus, cells expressing TurboGFP express proportionally the silencing RNA hairpin. We used two shRNA constructs that target mRNA coding mGluR1 homologous to rat, and for control, we used nonsilencing lentiviral vector with a scrambled sequence (Supplemental Experimental Procedures). Cultured GCs were infected on 0 DIV with multiplicity of infection equal 1 and were used for electrophysiology on 7 DIV. To control for mGluR1 expression, we fixed cells 10 min at room temperature (RT) with 4% paraformaldehyde, washed with PBS, permeabilized for 7 min in 0.1% Triton X-100 in PBS, and blocked for 1 hr at RT with 10% Normal Goat Serum in PBS. After blocking cells were incubated overnight with rabbit anti-mGluR1 antibody (Abcam; #ab82211), they were washed and incubated with fluorescent (Alexa 568) secondary anti-rabbit antibody (Invitrogen) for 40 min at RT, washed, and mounted.

### Monte Carlo Model: Synaptic Environment

Computations were carried out using an ad hoc built in-house 64-node PC cluster optimized for parallel computing (Zheng et al., 2008). The modeling Monte Carlo algorithms were adapted from our previous studies (Savtchenko and Rusakov, 2007; Sylantyev et al., 2008). Geometry of MF-GC synapses was approximated by the pre- and postsynaptic cuboid shapes (Figure 1C), adapting the structure of cerebellar glomeruli described previously by Nielsen et al. (2004). A total of 3,000 glutamate molecules were released in the center of the 600-nm-wide apposition area that was separated by a 50 nm space from neighboring structures (Figure 1C); the synaptic cleft height at the MF-GC interface was 16 nm, and the PSD was 160 nm wide. In most simulation experiments, 125 AMPARs and 50 NMDARs (available receptors) were scattered inside the PSD, with the channel conductance of 10 and 25 pS, respectively. Group I mGluRs were distributed on the PSD periphery (15-nm-wide ring, diameter 360 nm).

### Monte Carlo Model: Glutamate Electrodiffusion

Again, we adapted our earlier approach (Sylantyev et al., 2008) incorporating electric interactions between charged glutamate and receptor-generated currents. Diffusion therefore included (1) Brownian displacement $\Delta_{\text{b}}=\sqrt{6Ddt}$, where *dt* is the elementary time step, and *D* is the diffusion coefficient); and (2) displacement in the *XY* plane due to electric interactions inside the cleft. Electrical interactions were calculated from the relationship for the particle speed in the electric field dr/dt=−μE and mobility μ=Dq(F/RT), where vector **E** is the voltage gradient, **r** is the coordinate vector (*r* is thus the radial coordinate), *q* = −1 for glutamate, *F* is Faraday’s constant, *R* is the gas constant, and *T* is absolute temperature. In conditions of rotational symmetry and steady-state approximation (where spatial relaxation of the electrical field is much faster than diffusion), the radial voltage profile in the cleft follows the expressions (Savtchenko et al., 2000; Savtchenko and Rusakov, 2007):

$$V\left(r\right)=V_{o}\frac{I_{0}\left(r/\lambda \right)}{I_{0}\left(L\right)+LI_{1}\left(L\right)\;\ln\left(R/r_{a}\right)},\;r_{a}\;>\;r\;>\;0$$

$$V\left(r\right)=V_{o}\frac{I_{0}\left(L\right)+LI_{1}\left(L\right)\;\ln\left(r/r_{a}\right)}{I_{0}\left(L\right)+LI_{1}\left(L\right)\;\ln\left(R/r_{a}\right)},\;R\;>\;r\;>\;r_{a},$$

where *I* is the modified Bessel function, $L=\sqrt{\gamma NP\left(r\right)R_{\text{ex}}/\pi \delta }$, δ is the cleft height, $\;\lambda =r_{a}/L$, *V*_o_ is the resting membrane voltage outside the cleft, and γ stands for the single receptor conductance (see Supplemental Experimental Procedures for further details).

### Monte Carlo Model: Receptor Activation

The model duty cycle following glutamate release event was as described previously (Sylantyev et al., 2008). In brief, at each time step (0.1 μs), the model calculated the (1) coordinates of glutamate molecules, (2) concentration profile of glutamate *C*(*r,t*), and (3) average occurrence of open AMPARs and NMDARs [*O*](*r*) within the active zone (*r* < *r*_a_) in accordance with the kinetic schemes reported by, respectively, Jonas et al. (1993) and Lester et al. (1993). When γ-DGG was present, the AMPAR activation kinetics were computed according to Wadiche and Jahr (2001). These calculations gave the total synaptic current (Supplemental Experimental Procedures), which was used to compute molecular electrodiffusion (displacement) during the time step, thus, initiating the next duty cycle; the procedure was repeated throughout the model run. We routinely verified that reducing the time step 10-fold improved computation accuracy by <1%.

Model was adjusted for 33°C–35°C using Q_10_ ≈2 for the kinetics of NMDARs (Lester et al., 1993) and mGluR1s (Marcaggi et al., 2009) reported earlier for RT, which was in correspondence with the temperature adjustment in NEURON. Our patch experiments showed well-constrained adjustment for and good correspondence with the published AMPAR kinetics (Wadiche and Jahr, 2001), which was originally obtained for 33°C–35°C. The effective diffusion coefficient for glutamate *D*_*glut*_ varied from 0.25 μm^2^/ms inside the immediate cleft (packed with macromolecules) and 0.4 μm^2^/ms outside (inside the glomerula), in accord with the detailed experimental estimates of *D*_*glut*_ for these synapses (Nielsen et al., 2004).

### Monte Carlo Model: Postsynaptic Spikes

To reproduce the AP waveform, the postsynaptic membrane potential *V*_*m*_ was modeled as a time-dependent dynamic process that corresponded to the Hodgkin-Huxley membrane excitability model (Supplemental Experimental Procedures), which fits the dendritic AP waveform generated by the NEURON model of a GC (Figure S7B).

### Statistical Tests

Data were shown as mean ± SEM unless specified otherwise. We routinely used the t test (independent or paired sample) or nonparametric Wilcoxon test (when the data scatter deviated from the normal distribution). Scatter normality was examined using either direct comparison with the Gaussian or the *Z* scores.

## Supplemental Information

Document S1. Figures S1–S7 and Supplemental Experimental Procedures
