# Supplementary material for: Spike-Driven Glutamate Electrodiffusion Triggers Synaptic Potentiation via a Homer-Dependent mGluR-NMDAR Link
Source: Neuron. 2013 Feb 6;77(3):528–41. doi: 10.1016/j.neuron.2012.11.026 (PMC3568920; doi:10.1016/j.neuron.2012.11.026)
Supplement: Document S1. Figures S1–S7 and Supplemental Experimental Procedures [file mmc1.pdf]

Neuron, Volume *77*

Supplemental Information

Spike-Driven Glutamate Electrodiffusion

Triggers Synaptic Potentiation

via a Homer-Dependent mGluR-NMDAR Link

Sergiy Sylantyev, Leonid P. Savtchenko, Yaroslav Ermolyuk, Piotr Michaluk, and Dmitri A. Rusakov

SUPPLEMENTAL INFORMATION

SUPPLEMENTAL FIGURES

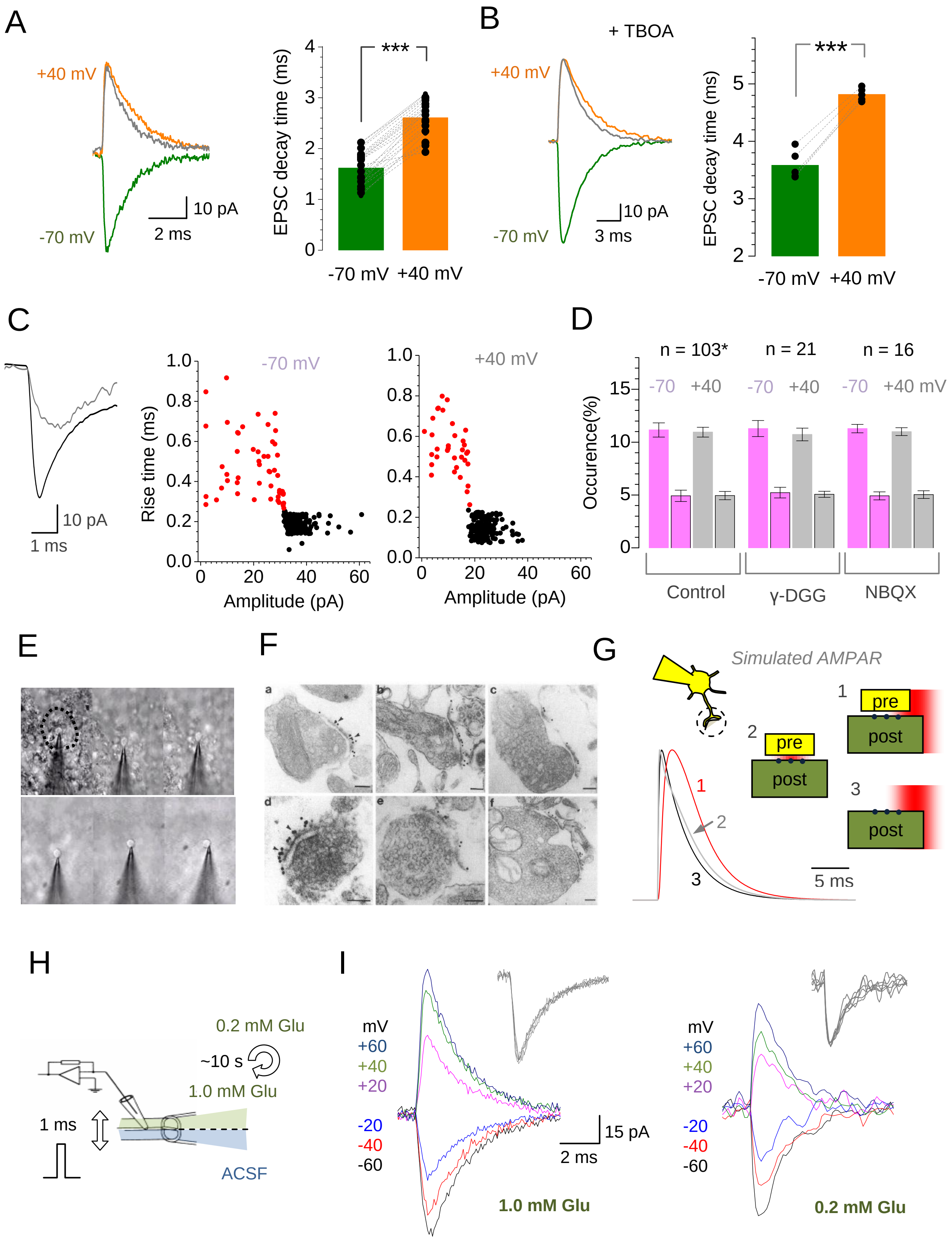

Figure S1. Probing the properties of EPSCs recorded at MF-CGC synapses.

(A) Traces, characteristic MF-evoked EPSCs recorded in CGCs at holding voltage V_h = -70 mV (green) and +40 mV (orange); gray, EPSCs at -70mV reversed and re-scaled. Graph, statistical summary; dots, individual experiments; columns, mean values; *** $p < 0.001$.

(B) Voltage asymmetry of the AMPAR-dependent EPSC decay remains intact under blockade of glutamate uptake by 50 µM TBOA. *** $p < 0.005$; other notations as in (A). Note that the EPSC decay is slower than that in control conditions, most likely due to retarded clearance of glutamate from the cleft.

(C) Traces: AMPAR EPSCs at MF-CGC synapses can be readily separated into larger, fast-rising (generated by glutamate released at the immediate synapse, black trace) and smaller, slow-rising (generated by glutamate that escapes from a neighboring glomerular synapse, gray trace), as reported previously (Nielsen et al., 2004). Plots: a scatter of EPSC rise times and amplitudes recorded in one example cell, at two holding voltages, as indicated; traces can be readily separated into spillover (red) and non-spillover (black) EPSC groups. mGluRs, $GABA_A$ and $GABA_B$ receptors are blocked.

(D) The proportion of spillover EPSCs (columns with no border) and failures (black bordered columns) remains unchanged upon voltage reversal, in 0.1 µM NBQX or 4 mM γ-DGG, as indicated, which partially block AMPARs (see experiments documented in Figure 2). The numbers of recorded cells are shown (*, 85 cell documented at +40 mV).

(E) The procedure to excise a CGC in whole-cell mode (further detail in Figure 1C) attempting to preserve a dendrite; a series of video snapshots depicts cell patching, pulling and lifting above the slice (right bottom image also depicts the patch exposed to the solutions flow from the θ-glass pipette); dotted line indicates where the slice tissue was loosened with a second pipette.

(F) Synaptic clefts are likely to remain attached to presynaptic terminals after mechanical separation of cells: example single-section electron micrographs (modified from (Hunt et al., 1996), with permission; rat hippocampus) in which immunogold labelled PSD-95 protein is documented in intact PSDs attached to synaptosomes that have been isolated via centrifugation. Scale bars, 100 nm.

(G) Simulated AMPAR currents (traces) that correspond to case 1-3 shown by inset diagrams: (1) a 1 ms pulse of 1 mM glutamate applied outside the cleft; (2) glutamate released in the synaptic cleft center; and (3) a 1 ms pulse of 1 mM glutamate applied

outside with no presynaptic fragment attached, as indicated; glutamate pulse is depicted by red shade. Top inset diagram: experimental schematic. The synaptic environment and the algorithms for glutamate diffusion and AMPAR kinetics in these simulations were the same as is described later in the text and Experimental Procedures (also Figure 1F).

(H) A schematic of the fast-application (and fast solution exchange) experiment; one or both theta-glass channel solutions can be replaced within ~10 s.

(I) Experimental traces depicting AMPAR responses at different holding voltages, as indicated by color coding, to 1.0 mM and 0.2 mM glutamate pulses applied to the same outside-out patch pulled from a CGC (the same patch is also depicted in Figure 1D, marked "excised"); inserts, traces re-scaled to the same amplitude. See Figure 1E for a statistical summary.

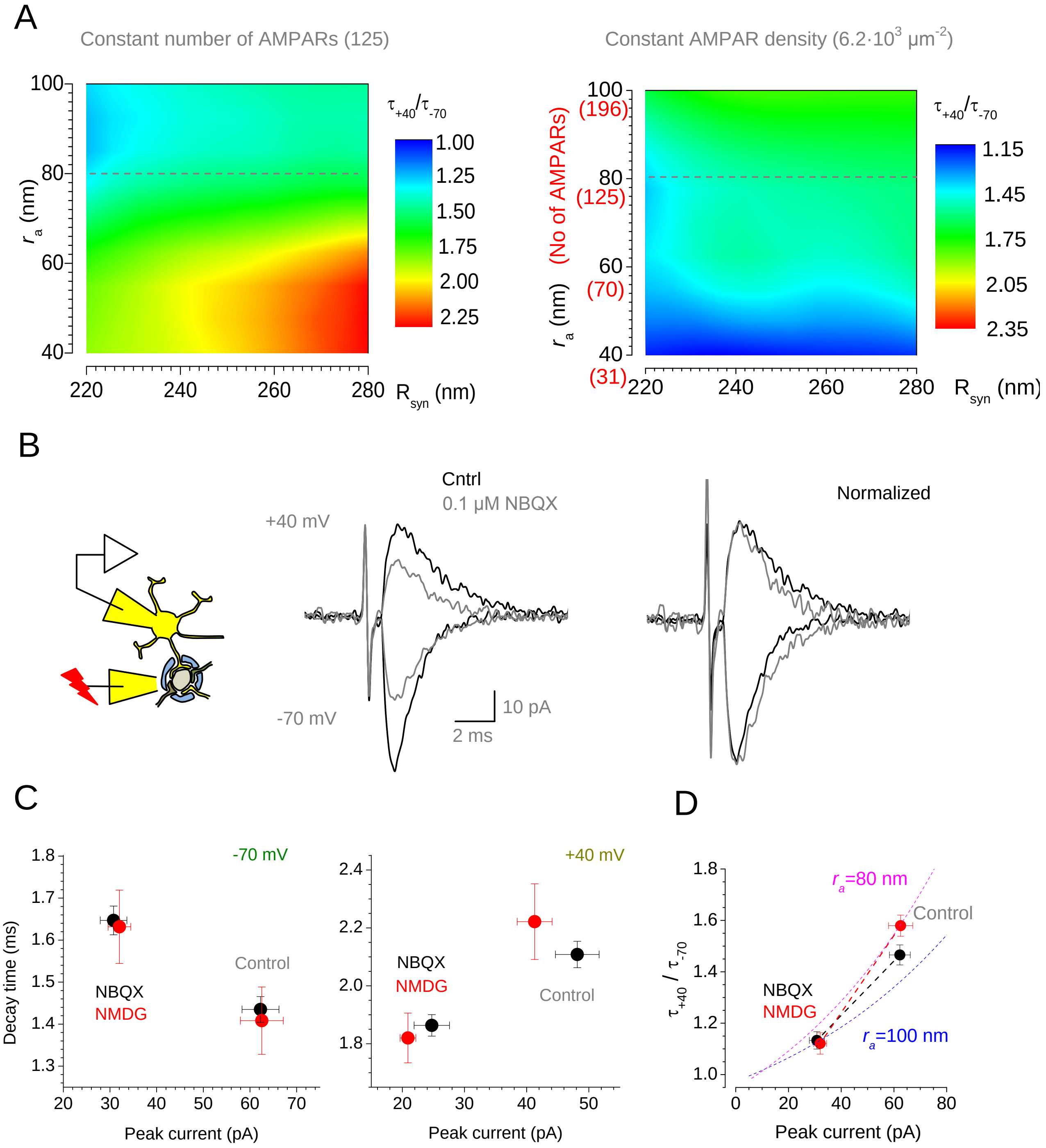

Figure S2. The extent of glutamate electrodiffusion depends on the numbers of available synaptic AMPARs.

(A) Simulated ratios of the EPSC decay times at +40 and -70 mV (τ_{+40} / τ_{-70}; color scale as shown) for different sizes of the synaptic apposition area (radius R_{syn}) and postsynaptic density size (equal to the active zone radius r_a) with either the total number or the average surface density of AMPARs remaining constant (left and right panels,

respectively). At r_a = 80 nm (average size, 125 AMPARs) the two map profiles are similar (dotted lines).

(B) Inset: experimental diagram (notations in Figure 1A). Traces, left panel: characteristic average EPSCs at -70 mv and +40 mV, before and after application of 0.1 μM NBQX, as indicated (one-cell example); right panel, traces in normalized to the same peak amplitude, illustrating that NBQX increases the EPSCs decay at -70 while decreasing it at +40 mV.

(C) Average effects of 0.1 μM NBQX and NMDG on the AMPAR EPSC peak amplitude and decay time, at -70 mV and +40 mV. Average peak amplitudes (abscissa) and decay times (ordinate) are shown (± SEM) for control and test conditions of the NBQX sample (black, $p < 0.001$ for the effect on decay time at both voltages, n = 15) and NMDG sample (red, $p < 0.005$ for the effect on decay time at both voltages, n = 6), as indicated.

(D) The relationships between the peak AMPAR current and the EPSC decay asymmetry (τ_{+40}/τ_{-70}). Dotted curves: prediction of the Monte Carlo model for two values of the active zone radius r_a, as indicated. Circles, average values (± SEM) for the NBQX (black) and NMDG (red) experimental samples, as indicated.

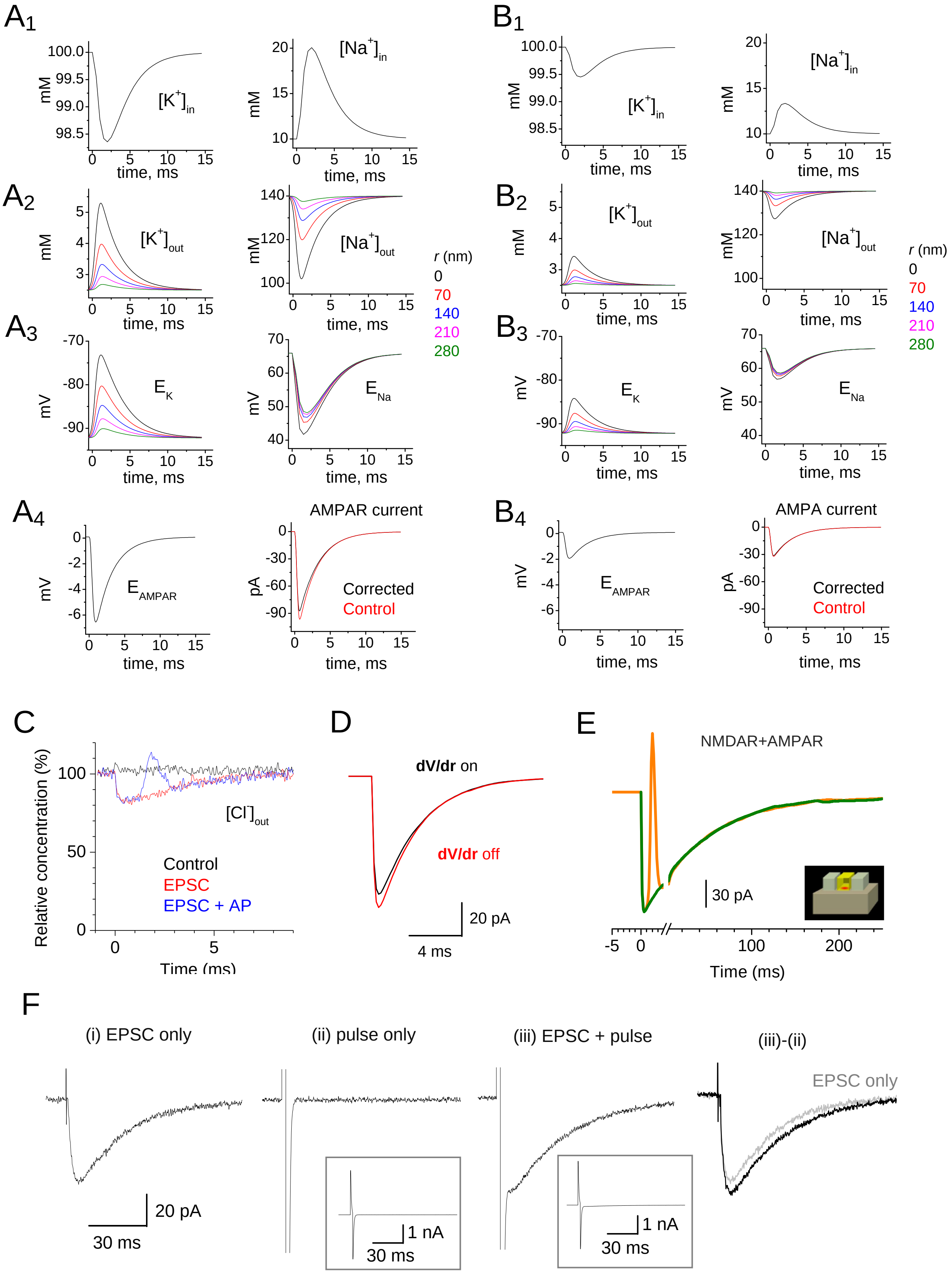
A1
[K+]in
[Na+]in
time, ms
B1
A2
[K+]out
[Na+]out
r (nm)
0
70
140
210
280
A3
EK
ENa
B2
B3
A4
EAMPAR
AMPAR current
Corrected
Control
B4
AMPA current
C
[Cl-]out
Relative concentration (%)
Control
EPSC
EPSC + AP
Time (ms)
D
dV/dr on
dV/dr off
20 pA
4 ms
E
NMDAR+AMPAR
30 pA
Time (ms)
F
(i) EPSC only
(ii) pulse only
(iii) EPSC + pulse
(iii)-(ii)
EPSC only
20 pA
30 ms
1 nA
30 ms

Figure S3. Exploring the effects of voltage-reversing spikes on receptor activation.

(A) Na^+ and K^+ dynamics inside the synaptic cleft and inside the postsynaptic dendritic "finger" during an EPSC and their effects on AMPAR activation: theoretical calculations for 300 available synaptic AMPARs (likely upper limit, peak open conductance 1360 pS). A_1: Time course of Na^+ and K^+ inside the postsynaptic "finger". The finger morphology was approximated with a 600 nm wide, 1 μm long cylinder connected to the (concentration-clamped) dendrite through a 60 nm wide 100 nm long neck; the latter was to mimic favorable conditions for local ion fluctuations, i.e. to enable a conservative / upper limit fluctuation estimate. Na^+ and K^+ concentrations in this two-compartment model were altered by EPSC-driven ion currents through AMPARs with the equilibration sink through the neck. $A_{2:}$ Time course of Na^+ and K^+ inside the synaptic cleft, at several distances from the cleft center, as indicated. The multi-compartmental model arena was 800 nm wide, with radial symmetry and concentrations clamped outside the 20 nm high cleft. Every time point (duty cycle) the concentration values were re-calculated taking account of the AMPAR current. A_3: The concurrent dynamics of the Na^+ and K^+ reverse potential (E_{Na} and E_K, respectively) inside the cleft, as indicated. A_4: The time course of the AMPAR reverse potential (E_{AMPAR}, left; calculated based on receptor permeability for Na^+, K^+ and Ca^{2+}, in accordance with (Mayer and Westbrook, 1987)) and AMPAR current (right), without ion fluctuation effects (black, Control) and corrected for the effect of current-evoked ion redistribution (red, Corrected); the overall effect is <10%.

(B) Computations similar to (A) but for 100 available synaptic AMPARs (peak open conductance 450 pS); notations as in (A).

(C) Monte Carlo simulations of Cl^- redistribution in the synaptic cleft during an EPSC and an action potential. 90000 negatively charged Cl^- ions were at t = 0 uniformly distributed inside the cleft and up to 300 nm outside the cleft. With Brownian motion enabled for all individual particles, the outer boundary condition with reflection was set, to maintain the global time-average concentration of Cl^- constant on a 100 ms scale. Individual molecules were traced during either an EPSC or action potential (onset at t = 0) generating a transient electric field in the cleft, as detailed earlier (Sylantyev et al., 2008) and in the Experimental Procedures. Diffusion coefficient was D = 0.6 μm^2/ms; traces show the concentration time course inside the active zone (R = 80 nm) in baseline steady-state conditions (black), and a concentration ripple during an EPSC (red) and an EPSC combined with a postsynaptic spike (blue).

(D) Monte Carlo simulated AMPAR current generated by glutamate release, with and without the effects of ion electrodiffusion inside the cleft (defined by radial voltage gradient, dV/dr) incorporated in the simulations, as indicated. See Experimental Procedures and earlier work (Savtchenko, 2007; Savtchenko et al., 2000; Savtchenko and Rusakov, 2007) for theoretical detail and model description.

(E) A postsynaptic AP has little effect on activation of synaptic AMPARs and NMDARs. Traces, simulated time course of EPSCs incorporating both AMPAR and NMDAR current components in baseline conditions (green) and with a postsynaptic AP (orange) generated, as indicated. The Monte Carlo modeling environment (inset) and parameters were as in Figures 3A and 3B.

(F) Three experimental traces routinely documented in voltage-jump experiments (one-cell example): (i) NMDAR EPSC, (ii) voltage-reversing (depolarizing) pulse, (iii) EPSC paired with the pulse, and a (iii)-(ii) trace subtraction (grey, control NMDAR EPSC trace shown in (i) panel).

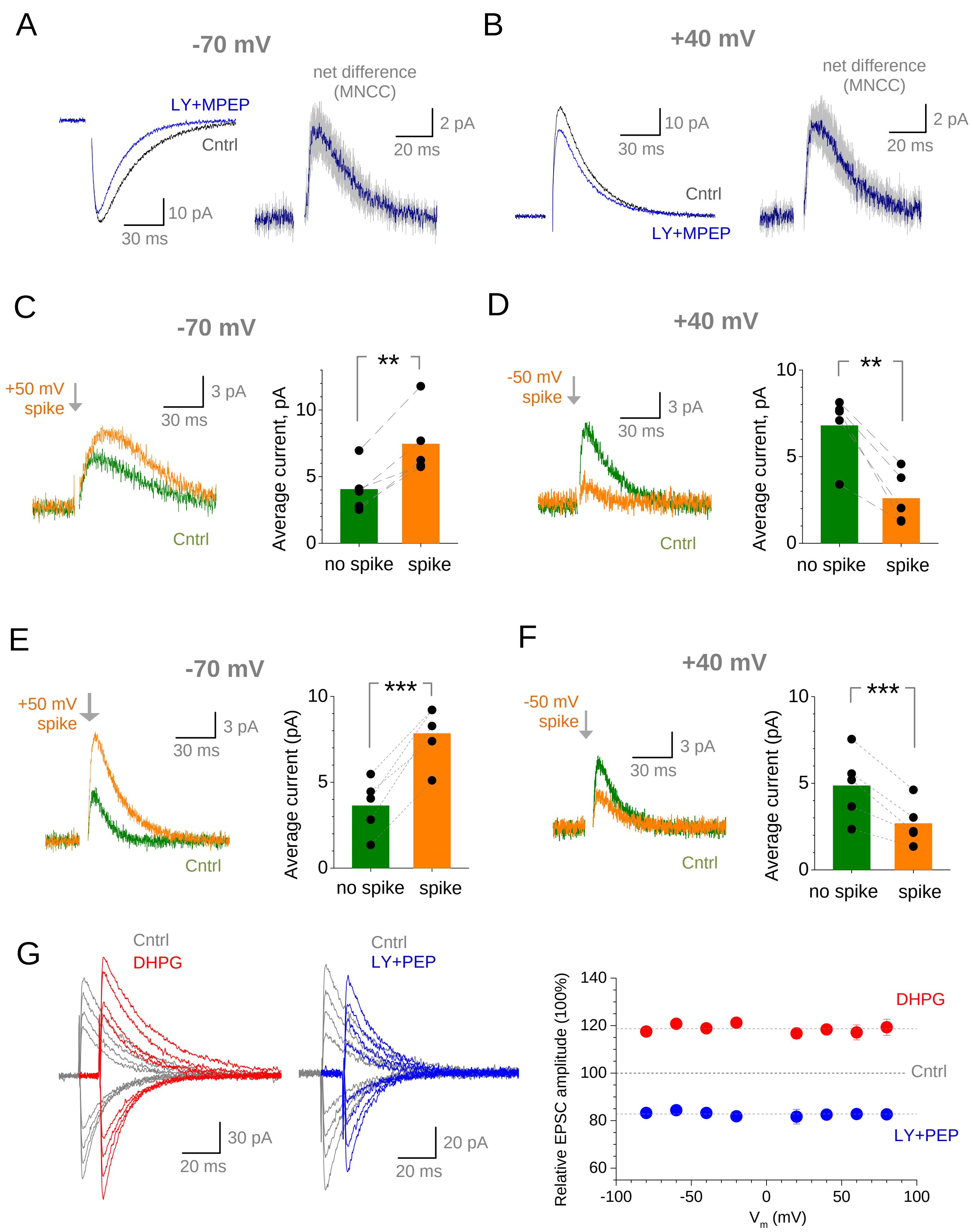

Figure S4. Spike-release pairing modifies the waveform of the mGluR -dependent component of NMDAR EPSCs, consistent with electrodiffusion of glutamate.

(A-B) The time course of the group I mGluR-dependent NMDAR current component (MNCC) at MF-CGC synapses. Black and blue EPSC traces, NMDAR-mediated EPSCs

evoked by MF stimulation with and without group I mGluRs blocked with LY367385 (100 μM) and MPEP (200 nM) at V_h = -70 mV (A) and +40 mV (B), as indicated (one cell example). In individual cells, the net difference between NMDAR EPSCs in the two conditions gives the MNCC time course. Dark blue traces and light blue shade, the global average MNCC ± 95% confidence intervals (n = 5).

(C-D) A postsynaptic spike coinciding with glutamate release at MF-CGC synapses increases the MNCC amplitude at -70 mV (C) while decreasing it at +40 mV (D); no appreciable effect on the MNCC duration is evident. Condition with and without spikes are color-coded in orange and green, respectively. Traces, characteristic recordings in one cell; Bar graphs, statistical summary: individual experiments (dots) and mean values (columns); ** $p < 0.01$.

(E-F) Blockade of mGluRs with the wide-range antagonist S-MCPG (200 μM) has the same effect as that of group I mGluR blockade (see Figure 4C). A postsynaptic spike coinciding with glutamate release (orange traces) increases the MNCC at -70 mV (E) while decreasing it at +40 mV (F) compared to control (green traces). Bar graphs, statistical summaries: dots, individual experiments; columns, mean values; ***$p < 0.005$.

(G) The effect on NMDAR EPSCs of either mGluR activation or mGluR blockade is voltage-independent. Traces, characteristic examples of NMDARs EPSCs, in control conditions (gray) and following application of either DHPG (red) or LY+MPEP (blue), recorded at different voltages between -80 mV and 80 mV, as indicated; traces are staggered for illustration purposes. Graph, statistical summary: average change in the NMDAR EPSC amplitude across holding voltages, as shown (mean ± SEM, n = 5).

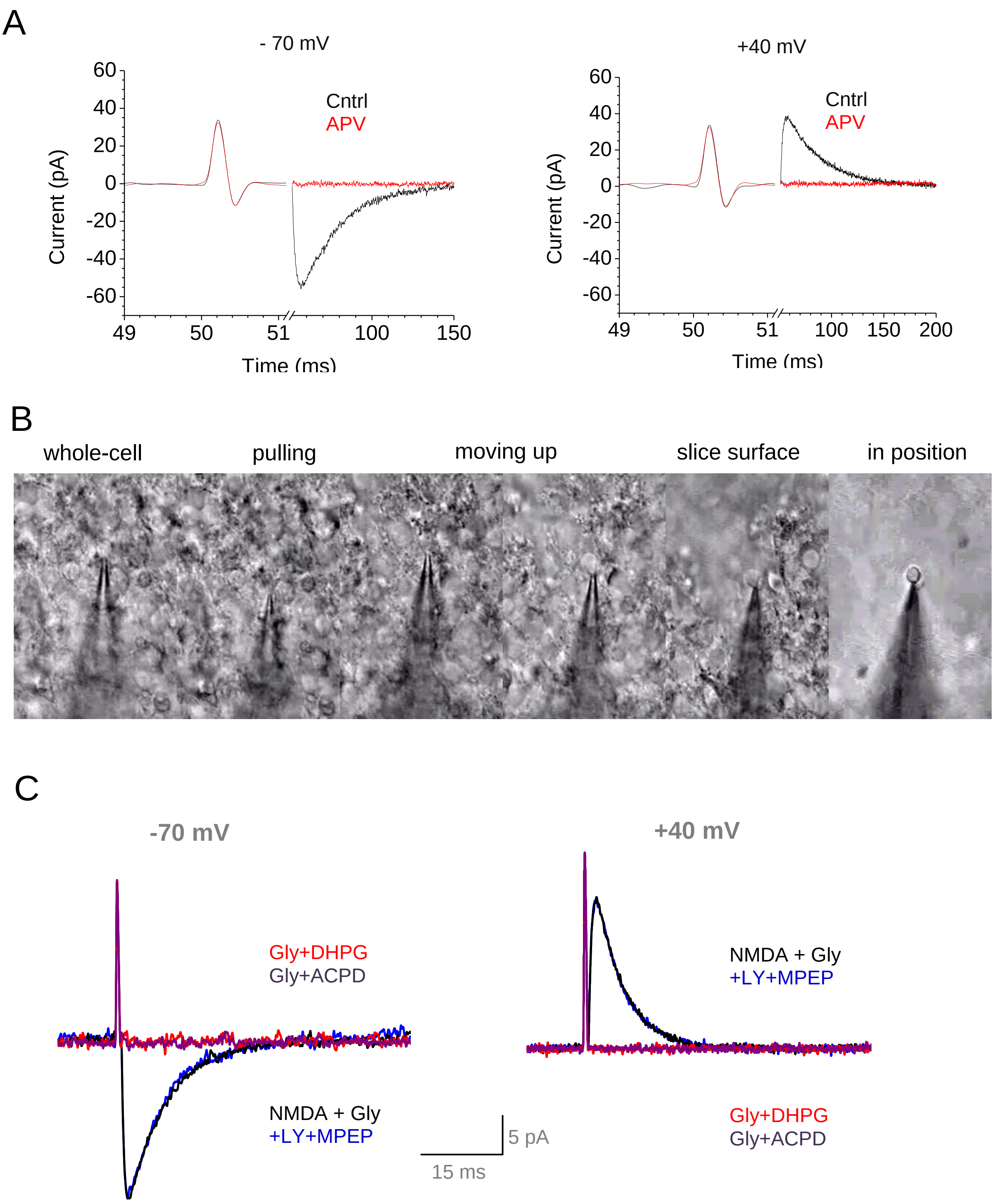

Figure S5. Probing voltage sensitivity of mGluR and NMDAR ligands.

(A) APV has no effect on the electrical stimulus deflection (artefact) at either negative or positive holding voltages. Characteristic one-cell example: traces of NMDAR EPSCs in control conditions (black) and following application of 50 μM APV (red) are shown using two different time scales, as indicated, at two holding voltages.

(B) A sequence of snapshots (differential inference contrast) illustrating experimental procedures to obtain a nucleated patch of a CGC for rapid ligand application probing. To achieve nucleated-patch configuration, we held the cell in whole-cell mode and then carefully pulled it from the slice so that the somatic membrane could stretch, close and tear behind the nucleus, as shown.

(C) Characteristic traces illustrating that NMDAR currents induced by ~1 ms pulses of 20 μM NMDA (black, in the presence of 1 mM glycine, Gly) are insensitive to blockade of mGluR1s with 0.1 mM LY367385 + 200 nM MPEP (blue) and that the application of mGluR agonists on their own (0.1 mM DHPG, red; 0.2 mM ACPD, purple) to the same patch produces no detectable response at either negative or positive holding voltage (n = 4 cells): one-patch example. Further details and statistical summary are shown in Fig. 4F.

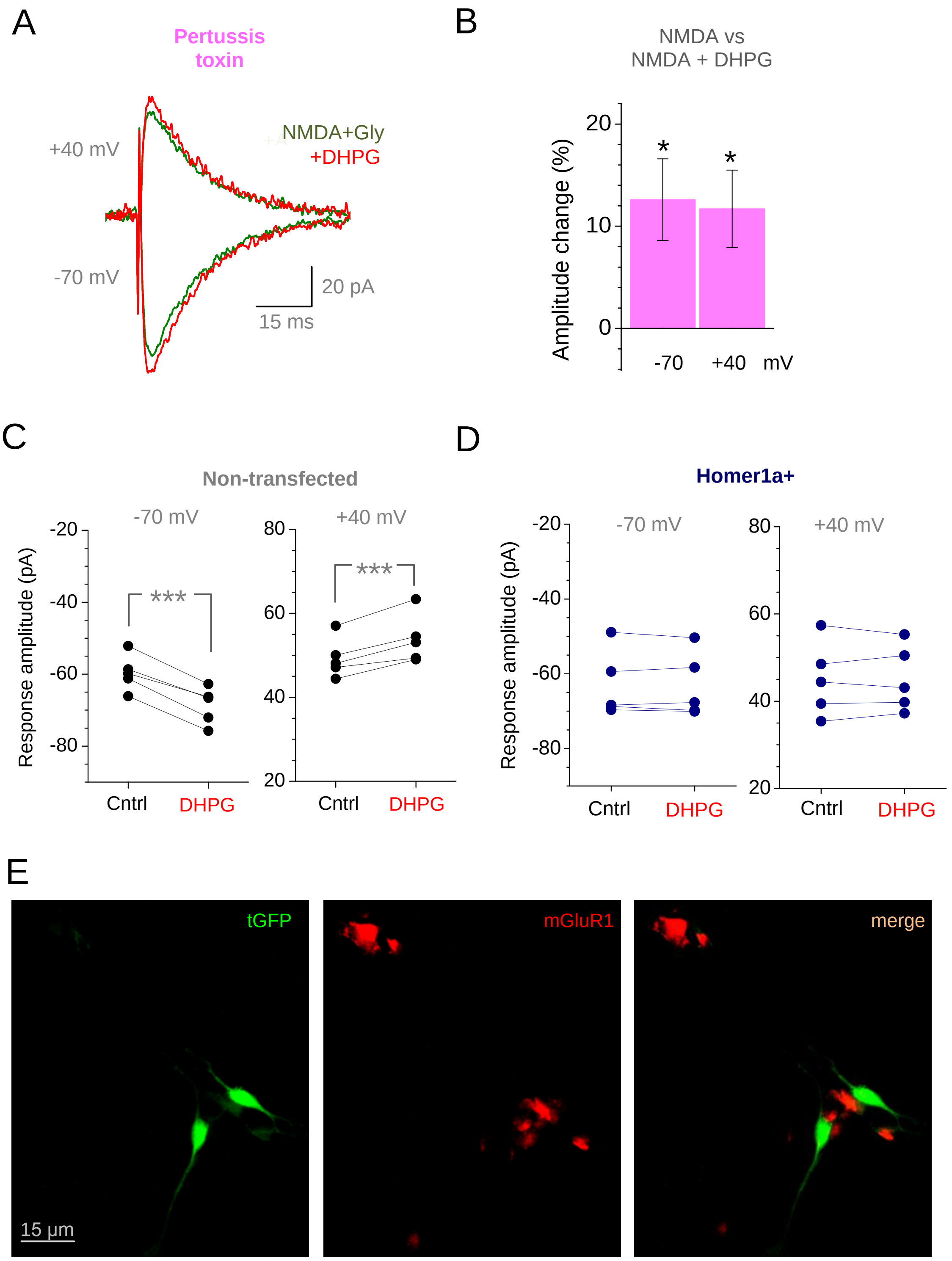

Figure S6. Molecular mechanisms underlying the facilitatory action of mGluRs on NMDA-induced NMDAR responses in CGC nucleated patches.

(A) NMDAR currents evoked in nucleated patches by 1 ms pulses of 0.2 mM NMDA are boosted by group I mGluR saturation with DHPG, at both negative and positive holding

voltages in the presence of pertussis toxin (right, 20 ng/ml) in the pipette: one-cell example.

(B) Statistical summary of experiments depicted in (A). Bars, average change (± SEM); * $p < 0.04$ ($n = 5$), as indicated.

(C) Activation of group I mGluRs with DHPG robustly increases the amplitude of NMDAR responses evoked by 1 ms pulses of NMDA in nucleated patches of non-transfected cultured CGCs, at both negative and positive holding voltages, as indicated; ***, $p < 0.005$.

(D) Application of DHPG has no effect on the evoked NMDAR responses in Homer1a-transfected CGCs in the same cultures, within a similar range of response amplitudes. See Figure 6 for further detail.

(E) Immunostaining control for mGluR1 gene silencing. Characteristic fluorescence images of cultured CGCs infected with a lentiviral vector coding turboGFP and shRNA against mGluR1. Left: tGFP positive cells (transduced with lentivirus; green), middle: localization of mGluR1 detected by a rabbit polyclonal antibody (red); right: merge tGFP and mGluR1 signal. It can be seen that expression of mGluR1 is suppressed in tGFP-positive cells. See Figure 6F-H for further data and Experimental Procedures for protocol details.

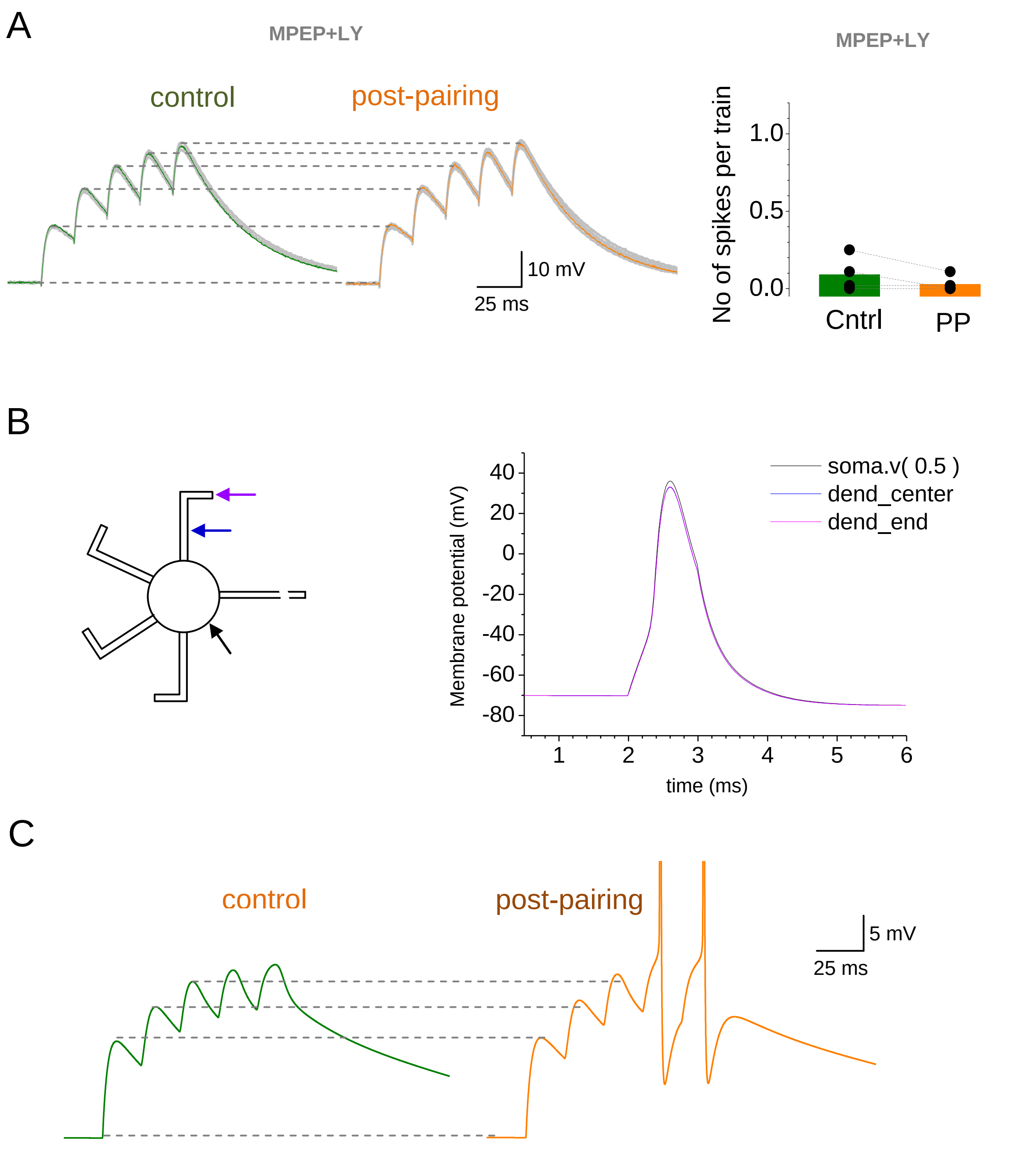

Figure S7. Spike-release pairing alters integrate-and-fire properties of the MF-CGC circuitry.

(A) Blockade of group I mGluRs blocks induction of potentiation induced by spike-release pairing. Traces, characteristic AMPAR- and NMDAR-dependent EPSPs (current clamp) evoked in a CGC by five MF stimuli before and after spike-release pairing, as indicated; green and orange lines depict an individual trace for comparison; gray lines show 4-6 consecutive traces; dotted lines depict summation of consecutive responses before and after pairing. Bar graph, summary: the average number of spikes per train

before and 3-4 min after pairing, as indicated (0.095 ± 0.057 and 0.033 ± 0.026, respectively, n = 4, $p > 0.8$).

(B) The NEURON database (Hines et al., 2004) model of a CGC (ModelDB Accession: 116835) was adapted from (Diwakar et al., 2009) with all the original parameters (there was no visible difference in traces between the original model temperature of 30C and when adjusted for 33C). Diagram on the left shows the adapted model cell morphology; arrows, recording sites for an action potential. Graphs, the waveform of spikes recorded at the corresponding sites, as indicated, suggesting a negligible difference between somatic and dendritic spikes in this compact cell.

(C) To replicate experimental setting (A) in the model shown in (B), five presynaptic discharges were set to occur 20 ms apart. The total conductance of the NMDARs and AMPARs were 1520 and 190 nS, respectively. In control conditions (left, green) the time constants of NMDAR kinetics were τ_1 = 2.79 ms, τ_2 = 13.3 ms, τ_3 = 103 ms (see Experimental Procedures for notations). These parameters were increased by ~20% post-pairing (orange, right), in accord with our experimental observations giving τ_1 = 3.50 ms, τ_2 = 16.6 ms, τ_3 = 129 ms: this EPSP slowdown post-pairing was due to an increased contribution of the relatively slow NMDAR-dependent component to the combined AMPAR+NMDAR synaptic response (see Figure 7A and 7D).

SUPPLEMENTAL EXPERIMENTAL PROCEDURES

Abbreviations

AMPA, α-amino-3-hydroxy-5-methylisoxazole-4- propionic acid; AMPAR, AMPA receptor; NMDA, N-methyl-D-aspartic acid; GABA, γ-aminobutyric acid; NMDAR, NMDA receptor; mGluR, metabotropic glutamate receptor; NBQX, 2,3-dihydroxy-6-nitro-7-sulfamoyl-benzo[f]quinoxaline-2,3-dione; D-APV, D-2-amino-5-phosphonovaleric acid; S-MCPG, α-methyl-4-carboxyphenylglycine; γ-DGG, γ-D-glutamylglycine; QX-314, N-(2,6-Dimethylphenylcarbamoylmethyl) triethylammonium bromide; MPEP, 2-Methyl-6-(phenylethynyl)pyridine hydrochloride; CGP-55845, (2S)-3-[[(1S)-1-(3,4-Dichlorophenyl) ethyl]amino-2-hydroxypropyl](phenylmethyl)phosphinic acid hydrochloride, LY 367385, (S)-(+)-α-Amino-4-carboxy-2-methylbenzeneacetic acid.

Electrophysiology: in situ

250 µm parasagittal slices were cut from the cerebellar vermis of 25-30 days old Sprague-Dawley rats and incubated for one hour in a solution containing (in mM): 124 NaCl, 3 KCl, 1 $CaCl_2$, 3 $MgCl_2$, 26 $NaHCO_3$, 1.25 NaH_2PO_4, 10 D-glucose, and bubbled with 95:5 O_2/CO_2, pH 7.4. After incubation slices were transferred to a recording chamber continuously superfused with an external solution. The external solution composition differed from incubation solution in containing 2 mM $CaCl_2$ and 2 mM $MgCl_2$ for AMPAR EPSC recording, and 2 mM $CaCl_2$, with zero Mg^{2+} for NMDAR EPSC recording. AMPAR EPSCs were isolated by addition of 1 µM CGP- 55845, 100 µM D-APV, and 100 µM picrotoxin. NMDAR-mediated EPSCs were isolated by addition of 1 µM CGP- 55845, 10 µM NBQX, and 100 µM picrotoxin. Where required, mGluR1s were blocked with 100 µM LY367385 and 200 nM MPEP applied together (or 200 µM S-MCPG where indicated). The intracellular pipette solution for voltage-clamp recordings contained (mM): 117.5 Cs-gluconate, 17.5 CsCl, 10 KOH-HEPES, 10 BAPTA, 8 NaCl, 5 QX-314, 2 Mg-ATP, 0.3 GTP; for current clamp: 126 K-gluconate, 4 NaCl, 5 HEPES, 15 glucose, 1 MgSO4*7H2O, 2 BAPTA, 3 Mg-ATP; (pH 7.2, 295 mOsm in both cases). Patch-clamp recordings were performed from GCs in the granular layer at 33-35ºC using Multiclamp-700B amplifier, in whole-cell mode; signals were digitized at 10 kHz. The pipette resistance was 7-9 MOhm. Capacitance and resistance properties of the cells were determined directly from amplifier's settings: series resistance 19.3±0.9 MOhm and membrane capacitance 4.7 ± 0.9 pF (n = 47) consistent with previous

measurements of GCs (Wyllie et al., 1993). MF axons were stimulated with a bipolar tungsten electrode placed in the cerebellar white matter near the gyrus crest to stimulate MFs entering the granule cells layer (Garthwaite and Batchelor, 1996). In the analyses of AMPAR EPSCs, smaller and slower "spillover" EPSCs and release failures (Figure S1C and S1D) were excluded from consideration.

Electrophysiology: rapid ligand application in outside-out and nucleated patches

Outside-out or nucleated patches were excised from cerebellar granule cells held in whole-cell mode, by slowly pulling the patch pipette to form a seal, as illustrated in Figures S1E and S5B. The fast ligand application method was adapted from (Colquhoun et al., 1992). We used a θ -glass application pipette pulled out to a ~200 μm tip diameter. The pipette was fixed in a micro-clamp, which was glued directly on a piezo bending actuator mounted on an electrode holder. Pipette channels were filled with the bath solution or bath solution containing different pharmacological agents (Figure 5A). Three separate micro-capillaries inserted into each of two channels provided application solution supply; a solution in each channel could be replaced within ~10 s by toggling the pressure pump circuit between the supplying micro-capillaries. Pressure in the application pipette channels was adjusted using the two-channel PDES-02DX pneumatic micro ejector (npi electronic GmbH) using compressed nitrogen. The ~1 ms electric pulses were applied via a constant voltage stimulus isolator; stimulus duration and amplitude were adjusted using a control test in which one pipette channel was filled with distilled water and the current was recorded by an open patch pipette. The characteristic time constant of the rapid-switch response in these control experiments was 150-250 μs, as documented earlier (Sylantyev et al., 2008). Nucleated and membrane patches were held 100-150 μm above the slice surface, with 4-5 mm of the θ-glass pipette tip submerged in the perfusion chamber at 33-35C. Before pulling the patch, we routinely checked the temperature in the application streams by placing a micro- thermocouple (Cole-Palmer Type-K, straight-shaft microprobe, tip diameter ~100 μm, precision ±1C) in the double-barrel streams, near the future position of the patch: no deviation from 33-35C was detected.

Cell culture preparation

Primary dissociated cerebellar granule cell neuronal cultures were prepared using cerebellar tissue from six-day-old rat pups (P6), in line with earlier studies (Silver et al., 1996). Cerebellar neurons were plated on coverslips coated with poly-L-lysine and cultured in Basal Medium Eagle supplemented with 10% FBS, 25mM KCl, 2mM glutamine, 100U/ml penicillin, and 0.1mg/ml streptomycin. The cultures were maintained in a humidified incubator in 5% CO_2 at 37°C. To restrict glial cell growth, 10µM cytosine-β-d-arabinofuranoside was added to the cultures 24 h after plating. The cultures were used for experiments at 6-7 days in vitro (DIV).

Cell cultures: transfection with Homer1a and knocking down the mGluR1 gene

Primary culture of cerebellar granular neurons were transfected at 5 DIV with pRK5-Homer1a (kindly provided by Julie Perroy and Laurent Fagni) using Effecten reagent (Qiagen), in accordance with manufacturer's instructions. A plasmid carrying mCherry under *Synapsin* promoter was used as a fluorescent transfection marker in electrophysiological studies. mCherry and Homer1a were co-transfected at 1:2 ratio. Two days after transfection, whole-cell test recordings were made in mCherry-positive cells, with a control group from mCherry-negative cells. Data were collected from at least three different cultures.

To silence the mGluR1 gene, we have used Thermo Scientific Open Biosystems Human GIPZ Lentiviral shRNAmir library (institutional subscription). GIPZ is miR30 based vector which drives expression of hairpin RNA and turboGFP from the same RNA polymerase II promoter (CMV promoter), thus cell expressing turboGFP express proportionally the silencing RNA hairpin. We used 2 shRNA constructs which target mRNA coding mGluR1 homologous to rat. These were clones V2LHS_130875 (targeting sequence: CAGGGAATGCCAATTCTAA) and V3LHS_318325 (targeting sequence: AGGTTATACGGAAAGGAGA). For control we used non-silencing lentiviral vector with scrambled sequence: ATCTGCTTGGGCGAGAGTAAG. Cultured cerebellar granule neurons were infected on the day of plating cells (0 DIV) with multiplicity of infection equal 1 and were used for electrophysiology on 7 DIV. To control for mGluR1 expression in transfected and non-transfected cells, we fixed cells 10 minutes at RT with 4% paraformaldehyde, washed with PBS, permabilized for 7 minutes in 0.1% Triton X-100 in PBS and blocked for 1 hour at RT with 10% Normal Goat Serum in PBS. After blocking cells were incubated overnight with rabbit anti-mGluR1 antibody (Abcam #ab82211), then washed and incubated with fluorescent (Alexa 568) secondary anti-

rabbit antibody (Invitrogen) for 40 minutes at RT, washed and mounted. Fluorescent specimens were examined under epifluorescence microscope equipped with a 40x objective (Figure S6E).

Monte-Carlo model: main notations and symbols

R – radius of the synaptic apposition zone; *δ* – synaptic cleft height; *Q* – the number of released neurotransmitter molecules; *D* – effective diffusion coefficient of glutamate in the cleft; D_{free} – diffusion coefficient of glutamate in a free aqueous medium; *t* – time variable; *r* – radial distance from the cleft centre (variable); *N* – total number of receptors (AMPA or NMDA) within the active zone; r_a – radius of the synaptic active zone; *P*(*r*) – fraction of open receptors; *V*(*r*) – local membrane potential; R_{ex} – extracellular medium resistivity inside the synaptic cleft; R_{free} – resistivity of a free extracellular medium; I_{syn} – total synaptic current through open receptors; *γ* – conductivity of a single receptor-channel; V_o – the postsynaptic resting membrane potential outside the cleft; V_c – the receptor reversal potential; J_0 and J_1 – Bessel functions of the first kind; I_0 and I_1 – modified Bessel functions; *C*(*r,t*) – local glutamate concentration; *R* –gas constant, *T* – absolute temperature; *F* – Faraday's constant.

Monte Carlo model: synaptic environment

Computations were carried using an ad hoc built in-house 64-node PC cluster optimized for parallel computing (Zheng et al., 2008). The modeling methodology and computational Monte Carlo algorithms were adapted from our approach detailed previously with regard to CA3-CA1 hippocampal synapses (Sylantyev et al., 2008). Geometric features of MF-GC synapses were approximated by the pre- and postsynaptic cuboid shapes, as illustrated in Figure 1C adapting the structure of cerebella glomeruli described in a previously published model (Nielsen et al., 2004). 3000 glutamate molecules were released in the center of the 600 nm wide apposition area separated by a 50 nm space from neighboring structures (Figure 1C); the synaptic cleft height at the MF-GC interface was 16 nm, the postsynaptic density was 160 nm wide. In most simulation experiments, 125 AMPARs and 50 NMDARs (the numbers of all available receptors of which only a proportion were activated post-release) were scattered inside the postsynaptic density, with the channel conductance of 10 and 25 pS, respectively. Metabotropic glutamate receptors were distributed evenly on the

periphery of the postsynaptic density (a 15 nm wide ring, 360 nm in diameter). Movements of individual molecules including Brownian motion, the electric field influence, glutamate binding to individual receptor molecules, and receptor states following activation were computed with a time step of 0.1 μs (Sylantyev et al., 2008); further reduction of the time step by an order of magnitude improved computation accuracy by <1%.

Monte-Carlo model: glutamate electrodiffusion

Again, we adapted our earlier approaches (Savtchenko and Rusakov, 2007; Sylantyev et al., 2008). As before, the present model included electric interactions between charged glutamate molecules and receptor-generated currents. Diffusion of individual particles therefore included (a) a regular Brownian displacement $\Delta_b = \sqrt{6Ddt}$ (*dt* is the elementary time step and *D* is the diffusion coefficient) and (b) two additional displacement components in the *XY*-plane due to electric interactions, Δ_e^x and Δ_e^y, inside the cleft. These calculations were carried out based on cylindrical symmetry of the field (an error due to the cuboid shapes of the synaptic elements at 250-300 nm from the center was negligible). Derivations of electrical interactions were calculated from the classical relationship for the particle speed in the electric field $\frac{d\mathbf{r}}{dt} = -\mu\mathbf{E}$ and mobility $\mu = Dq\frac{F}{RT}$ where vector **E** is the electric field (voltage gradient), **r** is the coordinate vector (*r* is thus the radial co-ordinate), *q* = –1 for glutamate, *F* is Faraday's constant, *R* is the gas constant, and *T* is absolute temperature. In conditions of rotational symmetry, at each *i*th time step this expression yields:

$$\Delta_e^x = -\frac{qDE_i x_i dt}{r_i}\frac{F}{RT} \text{ and } \Delta_e^y = -\frac{qDE_i y_i dt}{r_i}\frac{F}{RT} \qquad \text{(E1)}$$

where $r_i = \sqrt{x_i^2 + y_i^2}$. To calculate $E = -\frac{dV}{dr}$ (*V* is the intra-cleft voltage), the model solves a modified cable equation for the intra-cleft field (Savtchenko et al., 2000):

$$\left(\frac{\partial^2 V}{\partial r^2} + \frac{1}{r}\frac{\partial V}{\partial r}\right) - \frac{g_s(r)\,R_{ex}}{\delta}(V - V_c) = 0\,. \qquad \text{(E2)}$$

Here R_{ex} stands for the intra-cleft medium unit resistance, V_c = 0 is the receptor reversal potential, and $g_s(r)$ is the density of active synaptic conductance within the narrow ring

of radius (r + dr), so that $g_s(r) = \dfrac{P(r)g_{\max}}{\pi(2rdr + dr^2)}$ where $P(r)$ is the average opening probability for the receptor channels inside the ring.

In accordance with classical physics, the value of R_{ex} can be related to the diffusivity of the corresponding ion species through the expression

$$R_{ex} = \frac{RT}{F^2 \sum_i D_i z_i^2 C_i} \qquad \text{(E3)}$$

where D_i, z_i, and C_i stand for the diffusion coefficient, valence and concentration of the ion species in question. In the common case of steady-state approximation (where spatial relaxation of the electrical field is much faster than diffusion), the radial voltage profile in the cleft follows the expressions (Savtchenko et al., 2000; Savtchenko and Rusakov, 2007):

$$V(r) = V_o \frac{I_0(r/\lambda)}{I_0(L) + LI_1(L)\ln(R/r_a)}, \qquad r_a > r > 0 \qquad \text{(E4a)}$$

$$V(r) = V_o \frac{I_0(L) + LI_1(L)\ln(r/r_a)}{I_0(L) + LI_1(L)\ln(R/r_a)}, \qquad R > r > r_a. \qquad \text{(E4b)}$$

where I is the modified Bessel function, $L = \sqrt{\dfrac{\gamma NP(r)R_{ex}}{\pi\delta}}$, δ is the cleft height, $\lambda = \dfrac{r_a}{L}$,

V_o is the resting membrane voltage outside the cleft, and γ stands for the single receptor conductance. Expressions (E4) assume zero reversal potential for the receptor in question.

Monte-Carlo model: receptor activation

The model duty cycle following glutamate release event was as follows. At each time step (dt = 0.1 µs), the model first updated the co-ordinates of all individual glutamate molecules that follow Brownian movement and electric field effects (if any). Next, it calculated the concentration profile of glutamate $C(r,t)$ in the cleft. In conditions of approximate rotational symmetry (again, cuboid shapes of synaptic elements at 250-300 nm from the center had a negligible effect on these calculations), this corresponded to $C(r,t) = N_\delta \left(2\pi r \delta \Delta r\right)^{-1}$, where N_δ stands for the number of glutamate molecules

occurring at time point t inside the flat cylindrical ring of height δ, width Δr and radius r. The average occurrence (concentration) of open receptors $[O](r)$ within the active zone ($r < r_a$) was then calculated for the same time point from the multi-stage AMPAR or NMDAR kinetic scheme, in accordance with, respectively, (Jonas et al., 1993) and (Lester et al., 1993) using (a) the immediate history of receptor states, (b) the local glutamate concentration $C(r,t)$, and (c) the average concentration of receptors in the active zone, $N(\pi r_a)^{-2}$. When the fast-dissociating antagonist γ-DGG was present in the extracellular medium, the AMPA receptor activation kinetics were computed according to (Wadiche and Jahr, 2001).

These calculations gave the total synaptic current in the continuous (integral) and discrete forms, respectively, as

$$I_{syn} = 2\pi \int_0^{r_a} (V(r) - V_c) g_s(r) r dr \quad \text{, and} \qquad \text{(E5a)}$$

$$I_{syn} = 2\pi \sum_{i=1}^{r_a/\Delta r} iV(r)\gamma^2(\Delta r)[O](r) \qquad \text{(E5b)}$$

where $r_a/\Delta r$ was calculated to the nearest integer and the profile of $V(r)$ was calculated according to (E4). The outcome of expressions (E5) was then used to calculate molecular electrodiffusion during the next time step thus initiating the next duty cycle. The procedure was repeated systematically throughout the model run. We routinely verified that reducing the time step did not change the outcome of simulations.

The model parameters were adjusted for 33-35°C using $Q_{10} \approx 2$ for the kinetics of NMDARs (Lester et al., 1993) and MGluR1s (Marcaggi et al., 2009) reported earlier for room temperature, which was in correspondence with the global temperature adjustment in NEURON; our patch experiments allowed well-constrained adjustment for (and showed good correspondence with) the published AMPAR kinetics (Wadiche and Jahr, 2001) which was originally obtained for 33-35C. The effective diffusion coefficient for glutamate D_{glut} varied from 0.25 μm^2/ms inside the immediate cleft (packed with receptors and other macromolecules) and 0.4 μm^2/ms outside (still inside the glomerula), in accord with the detailed experimental estimates of D_{glut} for these synapses (Nielsen et al., 2004). Because the reported diffusion retardation factor for small glutamate molecules in the cleft (compared to a free medium) should be similar to that of other small ions, the cleft resistivity R_{ex} in our simulations was scaled up by the

same factor, with respect to the free medium resistance R_{free} ~60 Ohm·cm (Savtchenko and Rusakov, 2007).

Monte-Carlo model: postsynaptic spikes

To reproduce the AP waveform, the postsynaptic membrane potential V_m was modeled as a time dependent dynamic process which corresponded to the Hodgkin-Huxley membrane excitability model

$$V_m(\mathrm{t}) = V_{resting} + A\left(1 + \mathrm{Exp}\left(-\frac{(\mathrm{t} - \mathrm{t}_1) - \mathrm{x_c} + 0.5\mathrm{w}_1}{\mathrm{w}_2}\right)\right)^{-1}\left(1 - \left(1 + \mathrm{Exp}\left(-\frac{(\mathrm{t} - \mathrm{t}_1) - \mathrm{x_c} - 0.5\mathrm{w}_1}{\mathrm{w}_3}\right)\right)^{-1}\right)$$

with parameters x_c = 1 ms (initiation onset), A = 146 mV (voltage theoretical upper limit, AP present) or 0 mV (steady-state, $V_m = V_{resting}$), and spike kinetic parameters w_1 = 0.67 ms, w_2 = 0.117 ms, w_3 = 0.269 ms obtained by fitting the above formula to the dendritic AP waveform generated by the NEURON model of a granule cell (Fig. S7B).

Statistical tests

Data were represented as mean ± SEM unless specified otherwise. We routinely used the *t*-test (either independent or paired-sample, as per experimental design) or non-parametric Wilcoxon test (when the data scatter showed a significant deviation from the normal distribution). Scatter normally was examined using either direct comparison with the Gaussian or the *z*-scores.

SUPPLEMENTAL REFERENCES

Colquhoun, D., Jonas, P., and Sakmann, B. (1992). Action of brief pulses of glutamate on AMPA/kainate receptors in patches from different neurones of rat hippocampal slices. J Physiol *458*, 261-287.

Diwakar, S., Magistretti, J., Goldfarb, M., Naldi, G., and D'Angelo, E. (2009). Axonal Na+ channels ensure fast spike activation and back-propagation in cerebellar granule cells. J Neurophysiol *101*, 519-532.

Garthwaite, J., and Batchelor, A.M. (1996). A biplanar slice preparation for studying cerebellar synaptic transmission. J Neurosci Methods *64*, 189-197.

Hines, M.L., Morse, T., Migliore, M., Carnevale, N.T., and Shepherd, G.M. (2004). ModelDB: A Database to Support Computational Neuroscience. J Comput Neurosci *17*, 7-11.

Hunt, C.A., Schenker, L.J., and Kennedy, M.B. (1996). PSD-95 is associated with the postsynaptic density and not with the presynaptic membrane at forebrain synapses. J Neurosci *16*, 1380-1388.

Jonas, P., Major, G., and Sakmann, B. (1993). Quantal components of unitary EPSCs at the mossy fibre synapse on CA3 pyramidal cells of rat hippocampus. J. Physiol. *472*, 615-663.

Lester, R.A.J., Tong, G., and Jahr, C.E. (1993). Interactions between the glycine and glutamate binding sites of the NMDA receptor. J. Neurosci. *13*, 1088-1096.

Marcaggi, P., Mutoh, H., Dimitrov, D., Beato, M., and Knopfel, T. (2009). Optical measurement of mGluR1 conformational changes reveals fast activation, slow deactivation, and sensitization. Proc Natl Acad Sci U S A *106*, 11388-11393.

Mayer, M.L., and Westbrook, G.L. (1987). Permeation and block of N-methyl-D-aspartic acid receptor channels by divalent cations in mouse cultured central neurones. J Physiol *394*, 501-527.

Nielsen, T.A., DiGregorio, D.A., and Silver, R.A. (2004). Modulation of glutamate mobility reveals the mechanism underlying slow-rising AMPAR EPSCs and the diffusion coefficient in the synaptic cleft. Neuron *42*, 757-771.

Savtchenko, L.P. (2007). Bilateral processing in chemical synapses with electrical 'ephaptic' feedback: a theoretical model. Math Biosci *207*, 113-137.

Savtchenko, L.P., Antropov, S.N., and Korogod, S.M. (2000). Effect of voltage drop within the synaptic cleft on the current and voltage generated at a single synapse. Biophys J *78*, 1119-1125.

Savtchenko, L.P., and Rusakov, D.A. (2007). The optimal height of the synaptic cleft. Proc Natl Acad Sci U S A *104*, 1823-1828.

Silver, R.A., Colquhoun, D., Cull-Candy, S.G., and Edmonds, B. (1996). Deactivation and desensitization of non-NMDA receptors in patches and the time course of EPSCs in rat cerebellar granule cells. J. Physiol. *493*, 167-173.

Sylantyev, S., Savtchenko, L.P., Niu, Y.P., Ivanov, A.I., Jensen, T.P., Kullmann, D.M., Xiao, M.Y., and Rusakov, D.A. (2008). Electric fields due to synaptic currents sharpen excitatory transmission. Science *319*, 1845-1849.

Wadiche, J.I., and Jahr, C.E. (2001). Multivesicular release at climbing fiber-Purkinje cell synapses. Neuron *32*, 301-313.

Wyllie, D.J., Traynelis, S.F., and Cull-Candy, S.G. (1993). Evidence for more than one type of non-NMDA receptor in outside-out patches from cerebellar granule cells of the rat. J Physiol *463*, 193-226.

Zheng, K., Scimemi, A., and Rusakov, D.A. (2008). Receptor actions of synaptically released glutamate: the role of transporters on the scale from nanometers to microns. Biophys J *95*, 4584-4596.
